# USP1 modulates hepatocellular carcinoma progression via the Hippo/TAZ axis

**DOI:** 10.1038/s41419-023-05777-1

**Published:** 2023-04-12

**Authors:** Dongyi Liu, Quanhui Li, Yifeng Zang, Xin Li, Zhongbo Li, Peng Zhang, Chang Feng, Penghe Yang, Jiayao Cui, Yanan Sun, Tian Wei, Peng Su, Xin Zhao, Huijie Yang, Yinlu Ding

**Affiliations:** 1 Department of General Surgery, The Second Hospital, Cheeloo College of Medicine, Shandong University, Jinan, Shandong Province P. R. China; 2 Department of Anaesthesiology, The Second Hospital, Cheeloo College of Medicine, Shandong University, Jinan, Shandong Province P. R. China; 3 Xinxiang Key Laboratory of Tumor Migration and Invasion Precision Medicine, School of Laboratory Medicine, Xinxiang Medical University, Xinxiang, 453003 Henan Province P. R. China; 4 Department of Pathology, Qilu Hospital, Cheeloo College of Medicine, Shandong University, Jinan, Shandong Province P. R. China

**Keywords:** Tumour biomarkers, Checkpoint signalling

## Abstract

Hepatocellular carcinoma (HCC) is one of the most lethal malignancies worldwide. The Hippo signaling pathway has emerged as a significant suppressive pathway for hepatocellular carcinogenesis. The core components of the Hippo pathway constitute a kinase cascade, which inhibits the functional activation of YAP/TAZ. Interestingly, the overactivation of YAP/TAZ is commonly observed in hepatocellular carcinoma, although the inhibitory kinase cascade of the Hippo pathway is still functional. Recent studies have indicated that the ubiquitin‒proteasome system also plays important roles in modulating Hippo signaling activity. Our DUB (deubiquitinase) siRNA screen showed that USP1 is a critical regulator of Hippo signaling activity. Analysis of TCGA data demonstrated that USP1 expression is elevated in HCC and associated with poor survival in HCC patients. RNA sequencing analysis revealed that USP1 depletion affects Hippo signaling activity in HCC cell lines. Mechanistic assays revealed that USP1 is required for Hippo/TAZ axis activity and HCC progression. USP1 interacted with the WW domain of TAZ, which subsequently enhanced TAZ stability by suppressing K11-linked polyubiquitination of TAZ. Our study identifies a novel mechanism linking USP1 and TAZ in regulating the Hippo pathway and one possible therapeutic target for HCC.

## Introduction

Hepatic cancer is one of the deadliest malignancies throughout the world, causing 900 thousand cases and 830 thousand deaths worldwide [1, 2]. Hepatocellular carcinoma accounts for 90% of hepatic cancers, and several factors, such as HCV infection and alcohol consumption, can give rise to the oncogenic process of HCC [3]. The detailed mechanism of HCC progression is still not clear, but it has been proven that several pathways are dysregulated in HCC, leading to malignant transformation of hepatocytes to hepatocellular carcinoma cells in humans [4]. Although several advances have been made in HCC therapy, the overall outcome of HCC is still unfavorable. It is necessary for basic studies to be performed to uncover novel therapeutic targets for HCC.

The Hippo pathway is evolutionally conserved and plays an essential role in suppressing hepatocyte proliferation and survival [5]. The key components of the Hippo pathway consist of serine/threonine protein kinases 4/3 (MST1/2), large tumor suppressor kinase (LATS), yes-associated protein (YAP) and WW domain-containing transcription regulator protein 1 (WWTR1; TAZ) [6]. Activation of the Hippo pathway leads to SAV1 binding to MST1/2, which facilitates MST activation. MST1/2 activation increases LATS1/2 phosphorylation and inactivates YAP/TAZ, since phosphorylated YAP/TAZ is sequestered in the cytosol for UPS (ubiquitin‒proteasome system)-mediated degradation [7]. Previous studies have established the link between dysregulation of the Hippo pathway and hepatocellular carcinogenesis [6]. For example, abnormal activation of YAP and TAZ was observed in HCC and was correlated with poor differentiation and shorter survival times in patients with HCC [8]. In addition, the functions of Hippo pathway components in controlling liver size and the oncogenic process of HCC have been extensively studied [9, 10]. For example, MST1/2 loss in Alb-Cre mice results in HCC formation, while both HCC and cholangiocarcinoma develop in NF2- or LATS1/2-deficient livers [11]. Collectively, these data indicate that abnormalities in the Hippo pathway could be the major driver of HCC; thus, targeting Hippo signaling could be a promising strategy for HCC clinical treatment.

However, the inhibitory phosphorylation cascade of the Hippo pathway is still functional in most HCC cases. Nevertheless, it is unclear why YAP/TAZ still overactivate transcription. Recent studies have revealed that several other types of posttranslational modifications, such as phosphorylation and ubiquitination, also impact YAP/TAZ function and Hippo signaling activity [7, 12]. According to the current knowledge, YAP/TAZ protein stability is tightly controlled by the balanced activity of several ubiquitin ligases and deubiquitinases [13–15]. Deubiquitinases, which cleave ubiquitin from substrates, mainly stabilize certain proteins and inhibit protein degradation. One possible strategy to reactivate the inhibitory function of Hippo signaling is to modulate the balance in polyubiquitination activity between E3 ubiquitin ligases and deubiquitinases in HCC. Based on this possibility, we performed a DUB siRNA screen and identified USP1 as the critical factor for Hippo signaling.

The USP1 (ubiquitin carboxyl-terminal hydrolase 1) gene was first reported in 1998, and the encoded protein contains approximately 785 amino acids [16]. The functional domain of USP1, which catalyzes the cleavage of ubiquitin from substrates, is located at the C-terminus [17]. USP1 was reported to participate in the DNA damage response [18] by facilitating the formation of DNA replication forks. In addition, USP1 was reported to promote cancer progression in models of several malignancies. For example, USP1 can stabilize the estrogen receptors and promote the growth of ER-positive breast cancer [19]. USP1 can stabilize the snail protein and induce chemotherapy resistance in platinum-resistant tumor models [20]. However, the molecular mechanism of USP1 in HCC remains elusive. Our siRNA screen revealed potential important roles of USP1 in Hippo/TAZ axis activity and HCC progression. USP1 facilitated TAZ function by inhibiting TAZ K11-linked polyubiquitination, which subsequently promoted HCC progression. Our study proposed a regulatory link between USP1 and the Hippo/TAZ axis and a promising target for treating HCC.

## Materials and methods

### Cell lines and cell culture

The human HCC cell lines HLF and Hep3B, as well as the human embryonic kidney cell line HEK293, were obtained from the American Type Cell Culture Collection (ATCC). All cell lines were cultured in DMEM (41965, Life Technologies) supplemented with 10% FBS (10270, Life Technologies) and 1% penicillin/streptomycin (C0222, Beyotime) at 37 °C in 5% CO2.

### Plasmids and siRNA transfection

The Myc-TAZ and TAZ deletion constructs and TEAD reporter plasmids were generated in our previous study [21]. The Flag-USP1 plasmids were a kind gift from Zhiguo Niu [19]. Transfection was performed using Lipofectamine 2000 (1662298, Invitrogen) according to the manufacturer’s instructions. The Lipofectamine RNAi MAX (Invitrogen 13778-075) transfection protocol was used for siRNA transfection after the cells were approximately 50% confluent. The small interfering RNAs (siRNAs; GenePharma) targeting USP1 with the following sequences were obtained. There were four sequences of USP1 siRNA used in this study: GUAUACUUCAGGUAUUAUAdTdT and UAUAAUACCUGAAGUAUACdTdT; CCAUACAAACAUUGGUAAAdTdT and UUUACCAAUGUUUGUAUGGdTdT. There were two negative control siRNA sequences: UUCUCCGAACGUGUCACGUTT and ACGUGACACGUUCGGAGAATT.

### Quantitative real-time PCR

Isolation of total RNA from cells was conducted according to the manufacturer’s instructions using TRIzol (A33250, Thermo), and reverse transcription was performed with HiScript II Q Select RT SuperMix (#R233-01, Vazyme). mRNA expression was measured with SYBR qPCR Master Mix (Q511-02, Vazyme) in an ABI Prism 7500 system. The primer sequences were as follows: TAZ 5′-CACCGTGTCCAATCACCAGTC-3′ (forward); 5′-TCCAACGCATCAACTTCAGGT-3′ (reverse). CTGF (CCN2): 5′-CAGCATGGACGTTCGTCTG-3′ (forward); 5′- AACCAC GGTTTGGTCCTTGG-3′ (reverse). CYR61 (CCN1): 5′-GGTCAA AGTTACCGGGCAGT-3′ (forward); 5′-GGAGGCATCGAATCCCAGC-3′ (reverse). USP1: 5′-GCTTTGCTGCTAGTGGTTTG-3′ (forward); 5′-GTTGGCTTTGTGCTCCATTC-3′ (reverse). 36B4: 5′-CGACCTGGAAGTCCAACTAC-3′ (forward); 5′-ATCTGCTGCATCTGCTTG-3′ (reverse). With the 36B4 as the control gene for normalization, analysis of the data was conducted using the 2-ΔΔCt method.

### Western blotting and coimmunoprecipitation assay

For Western blotting, cells were harvested and lysed with Western and IP Lysis Buffer (P0013J, Beyotime) containing protease and phosphatase inhibitors (Thermo). the Protein concentration was determined by Bradford protein assays. SDS-polyacrylamide gel electrophoresis (PAGE) was used to separate 20–30 µg of protein, and proteins were transferred to PVDF membranes (Millipore). The primary antibodies used were as follows: anti-USP1 (Cell Signaling Technology, Cat# 8033, 1:2000); anti-HA (Cell Signaling Technology, Cat# 3724, 1:2000); anti-Flag (Sigma–Aldrich, Cat# F9291, 1:2000); anti-TAZ (Abcam, ab224239, 1:2000); anti-Myc (Abcam, Ab9106, 1:1000); anti-Ki67 (Abcam, ab254123, 1:5000); anti-E-cadherin (Cell Signaling Technology, Cat# 4065, 1:5000); anti-N-cadherin (Proteintech, 22018-1-AP, 1:8000) and anti-β-actin (Sigma, A5441, 1:8000). PBST was used to wash the membranes three times before secondary antibodies were added (Beyotime, A0216, 1:5000 or Beyotime, A0208, 1:5000). ECL western blotting substrate was used to visualize the signals.

For coimmunoprecipitation assays, 500 µg of protein lysate was precleared with 20 ml Protein A + G Agarose (Beyotime, P2028) and rabbit IgG (Beyotime, A7016, 1:50) for 2 h at 4 °C, and immunoprecipitation was then performed with the indicated antibody for 4 h at 4 °C. As a negative control, either rabbit IgG (Beyotime, A7016, 1:50) or mouse IgG (Beyotime, A7028, 1:50) was used. Western blot analysis with the indicated antibodies was performed.

### Cell proliferation assay

A combination of 20 nM USP1 siRNA and 50 nM siControl was used to transfect HLF and Hep3B cells in 24-well plates. Twenty-four hours later, the cells were counted, and 4000 cells were seeded into 96-well plates. Then, the assays were performed as previously described [22].

### Clone formation assay

A combination of 20 nM USP1 siRNA and 50 nM siControl was used to transfect HLF and Hep3B cells in 24-well plates after 24 h. Trypsinized cells were plated at a low density (1000 cells per well) in a 6-well plate. of the cells were cultured for 2 weeks, during which the medium was refreshed every four days. Crystal violet was used to stain the colonies.

### Transwell assay

Corning 3422 inserts were used to evaluate the cell migration capacity. HLF and Hep3B cells were transfected with siUSP1 or siControl. Then, the assays were performed as previously described [22].

### Lentivirus production and lentiviral DNA constructs

Co-transduction of the lentiviral vectors with psPAX2 and pMD2.G was carried out with Lipofectamine 3000 (Invitrogen). After transduction for 36 h, medium containing viral particles was collected and passed through a 0.45 μM filter (Sigma). Infected HLF cells were incubated with the viral supernatant in the presence of 8 μg/mL polybrene (Beyotime). Stably transfected cells were selected with 2 μg/mL puromycin (Beyotime).

shUSP1 or shTAZ constructs were inserted into the PLVX-shRNA2-puro. The sequence of the shRNA targeting USP1 was 5′-CAGAGACAAACTAGATCAA-3′. The sequence of the shRNA targeting TAZ was 5′- AGGTACTTCCTCAATCACA -3′. The sequence of the nontargeting control shRNA (shControl) was 5′-CAACAAGATGAAGAGCACCAA-3′. The pLVX-USP1 were constructed by cloning an USP1 PCR fragment into the pLVX-EF1a-ZsGreen vector by T4 DNA ligase.

### In vivo tumorigenesis assay

Animals were maintained in a specific pathogen-free (SPF) environment with 12 h of light/12 h of darkness and free access to food and water. SPF (Beijing) Biotechnology Co., Ltd. provided female BALB/c nude mice for the tumor xenograft assay. Six-week-old female BALB/c nude mice were randomly divided into two or three groups and then injected with 3 × 10^6^ HLF cells in 150 μl PBS. The equation used to calculate tumor volume is volume = length × (width ^2^)/2.

### Dual-luciferase reporter assay

We measured the activity of the TEAD luciferase reporter by transfecting HLF and Hep3B cells expressing siUSP1 or siControl with the Renilla luciferase reporter and the TEAD luciferase reporter. A Dual-Luciferase Reporter Assay System (E1910, Promega, USA) was used to measure luciferase activity after 24 h.

### ChIP assay

ChIP was performed by using a modified protocol from the ChIP Assay Kt (17–295; Millipore). Suitable amounts of chromatin were immunopreciptated with specific antibodies overnight. Antibodies used were IgG (#026102; Life Technologies) and TAZ (#4883; Cell Signaling). Complexes were recovered on ChIP-grade Protein A/G plus agarose bead (#26195; Life Technologies). qPCR was performed using primer sets flanking the predicted TEAD binding sites. Primer sequences of CTGF used are as follows: 5′-TGTGCCAGCTTTTTCAGACG-3′; R 5′-TGAGCTGAATGGAGTCCTACACA-3′.

### Immunofluorescence assay

Coverslips were placed in the wells of 12-well cell culture plates containing HLF cells. After 24 h, HLF cells on the coverslips were fixed with 4% paraformaldehyde for 15 min, permeabilized with 0.25% Triton X-100 for 15 min, and blocked with 3% BSA for 1 h. The coverslips were incubated with primary antibodies against USP1 (Cell Signaling Technology, Cat# 8033, 1:200) and TAZ (Abcam, ab224239, 1:200) at 4 °C overnight. After washing with PBS, the coverslips were incubated with a fluorophore-conjugated secondary antibody (Invitrogen), and DAPI (Beyotime) was then used to stain nuclei. Samples incubated only with secondary antibodies and not with primary antibodies were used as the negative controls. Images were captured with a Nikon A + laser scanning confocal microscope. ImageJ was used to further process and assemble the acquired images.

### Cycloheximide assay

HLF cells were transfected for 24 h with siUSP1 or siControl. Then, 100 μmol/L cycloheximide (CHX) was added to the culture medium, and cell lysates were collected 0, 2, 4, 6, and 8 h after the addition of CHX. Flag-USP1 or Flag vectors were transfected into HEK293 cells. After 24 h, the cells were treated with CHX at a final concentration of 100 μmol/L, and lysates were harvested 0, 2-, 4-, 6-, and 8-h post-treatment.

### Polyubiquitination assay

To directly quantify the enrichment of ubiquitinated TAZ in cell extracts, Ub plasmids, Myc-TAZ vectors and Flag-USP1 or Flag vectors were transfected into HEK293 cells. After 24 h, the cells were treated with 10 μM MG132 for 8 h. Then, total protein was extracted, and 20 μL of protein A + G (Beyotime, P2012) was used to preclear the lysate for 2 h. The supernatant was harvested and immunoprecipitated with an anti-TAZ antibody. Western blotting was performed with an anti-HA antibody to detect polyubiquitination of TAZ.

### RNA sequencing and data analysis

In this study, RNA sequencing was performed by Beijing Genomics Institute (BGI) to analyze the expression of many genes (siControl and siUSP1). Data from the RNA sequencing analysis have been deposited in the Gene Expression Omnibus (GEO) database (accession number: GSE218366). Ingenuity Pathway Analysis (IPA) was performed on genes that were differentially expressed (*P* < 0.05 and fold change >2). For gene set enrichment analysis (GSEA), the GOBP_HIPPO_SIGNALING gene set was applied and was downloaded from the GSEA Molecular Signatures Database. GSEA 4.2.3 software was used to implement GSEA.

### Analysis of TCGA data and progression-free survival data

With Xiantao online (https://www.xiantao.love/), USP1 and TAZ mRNA levels were measured in normal liver tissues and hepatocellular carcinoma tissues of different stages. Via the KMPLOT (https://kmplot.com) online analysis database, progression-free survival (PFS) data were generated for patients based on their USP1 and TAZ expression levels.

### Statistical analysis

GraphPad Prism 9 software was used to conduct the statistical analyses. The results of a minimum of three independent experiments are presented as the means and standard deviations. We used two-tailed Student’s *t* test to assess the significance of differences. We considered a *P* value of at least 0.05 to indicate statistical significance.

## Results

### USP1 is an important regulator of Hippo signaling activity and exhibits elevated expression in human hepatocellular carcinoma

**Fig. 1:**
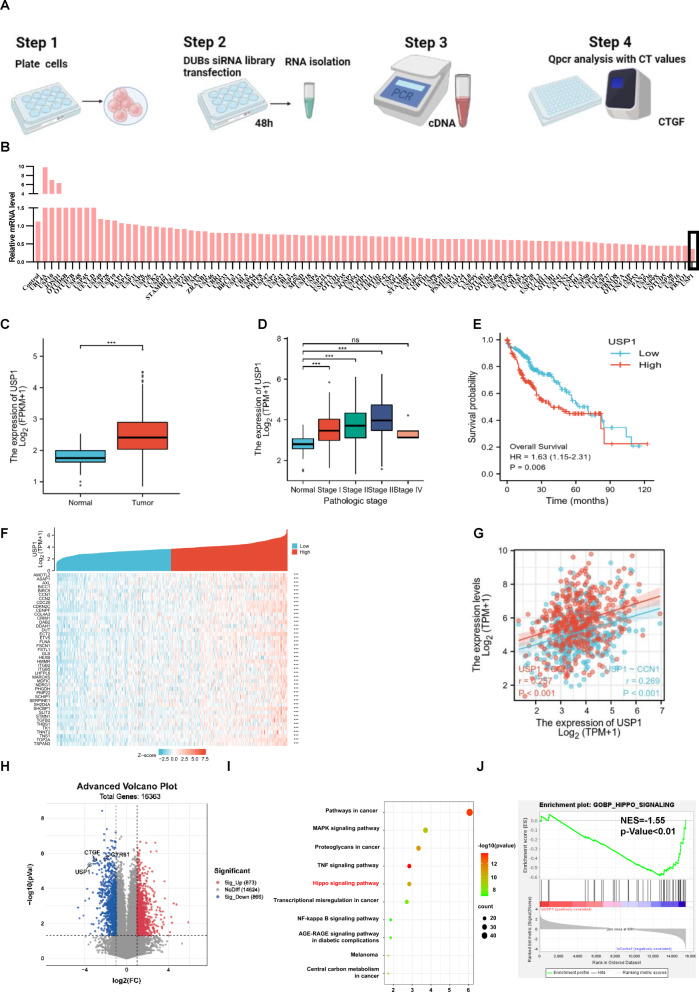
USP1 is an important regulator of Hippo signaling activity, and its expression is elevated in human hepatocellular carcinoma. **A** The flowchart shows the siRNA screening procedure for identifying novel deubiquitinases involved in modulating Hippo signaling. HEK293 cells were seeded in 24-well plates. Each well was transfected with a siRNA against one deubiquitinase and incubated for 48 h. RNA was extracted, and CTGF expression was assessed by qPCR. **B** In the siRNA screen of deubiquitinases, relative CTGF mRNA levels were determined. The red columns represent relative mRNA level of CTGF after depletion of USP1. **C** The relative RNA level of USP1 in HCC tumor samples (*n* = 371) versus normal samples (*n* = 50) in the TCGA database (https://portal.gdc.cancer.gov/). **D** The relative RNA level of USP1 in HCC tumors of different stages (Stag I, *n* = 168; Stag II, *n* = 84; Stag III, *n* = 82; Stag IV, *n* = 6) was compared with that in normal liver tissue (*n* = 50). Data were obtained from the TCGA database (https://portal.gdc.cancer.gov/). **E** Comparing with lower USP1 expression (*n* = 275), higher USP1 expression (*n* = 90) was associated with poorer overall survival in HCC patients based on Kaplan‒Meier analysis in TCGA. *P* < 0.001, log-rank test. **F** TCGA analysis of the correlation of USP1 expression with that of classical downstream genes of the Hippo signaling pathway in HCC (*n* = 371), with *p* < 0.001 as the threshold. **G** USP1 expression was significantly correlated with that of CCN1 (CYR61) and CCN2 (CTGF) in HCC (*n* = 371). **H** Volcano plot of RNA-seq data in HLF cell lines treated with siControl or siUSP1. The volcano plot revealed a significant increase in the expression of CTGF and CYR61 downstream of YAP as a result of USP1 depletion. Threshold values of P < 0.05 and fold change>2 was set as screening criteria. **I** These are the top 10 KEGG pathways that are significantly depleted (top) or enriched (bottom) in HLF cells treated with siUSP1. The pathway enrichment analysis consisted of differentially regulated genes identified with threshold criteria of P < 0.001 and fold change >2. The cells were treated for 48 h with 50 nM siUSP1 or vehicle. Total mRNA was extracted for RNA sequencing analysis. *n* = 3. **J** HLF cells treated with USP1 siRNA had depletion of Hippo pathway signature genes, according to gene set enrichment analysis (GSEA).

A DUB siRNA library (Dharmacon Company, Cat: G104705) containing 100 siRNAs specific for each DUB was used to screen for critical DUBs in the modulation of Hippo signaling. In view of the widespread usage of HEK293 cells in Hippo studies and the ease with which they can be transfected, we used them as our model. The expression of CTGF, the most classical Hippo target gene, was used as the readout. (Fig. 1A). A number of reported DUBs were identified in our screening assay, including OTUB1 and DUB1 (USP36), and we also identified USP1, whose expression also dramatically affected the mRNA level of CTGF (Fig. 1B). USP1 expression is reportedly elevated in many tumor tissues compared to normal tissues, including osteosarcoma and breast cancer tissues [23, 24]. We further investigated the expression of USP1 in hepatocellular carcinoma. Interestingly, analysis of the TCGA database indicated that the mRNA level of USP1 was elevated in HCC compared with normal liver tissues (Fig. 1C). Furthermore, USP1 was elevated in pathological stage I, II and III HCC tissues compared with normal hepatocellular tissue (Fig. 1D). In a further analysis of the TCGA database, high USP1 expression was associated with poor survival in HCC patients. (Fig. 1E). In addition, when we further analyzed the correlations of USP1 expression with that of classical downstream genes of the Hippo signaling pathway in the TCGA database, with *p* < 0.001 as the threshold, we found that USP1 had a significant positive correlation with 44 genes, including AMOTL2, ASAP1, CCN1 (CYR61), and CCN2 (CTGF), which exactly validated our DUB siRNA library screening results (Fig. 1F, G). Moreover, this conclusion was confirmed by RNA sequencing analysis in HLF cells (GSE218366). The volcano plot indicated that USP1 depletion significantly inhibited the expression of classical Hippo pathway target genes, including CYR61 and CTGF (Fig. 1H). In addition to the Hippo pathway, several other oncogenic pathways, such as the MAPK pathway, TNF pathway and NF-kappa B signaling pathway, were affected by USP1 depletion, according to KEGG pathway analysis (Fig. 1I). GSEA (gene set enrichment analysis) indicated that the GOBP_HIPPO_SIGNALING signature was significantly suppressed in the presence of USP1 depletion (Fig. 1J). Considering these results collectively, we speculate that USP1 could be a promising positive regulator of the Hippo signaling in human hepatocellular carcinoma.

### USP1 depletion suppresses Hippo/TAZ axis activity in HCC cells

**Fig. 2:**
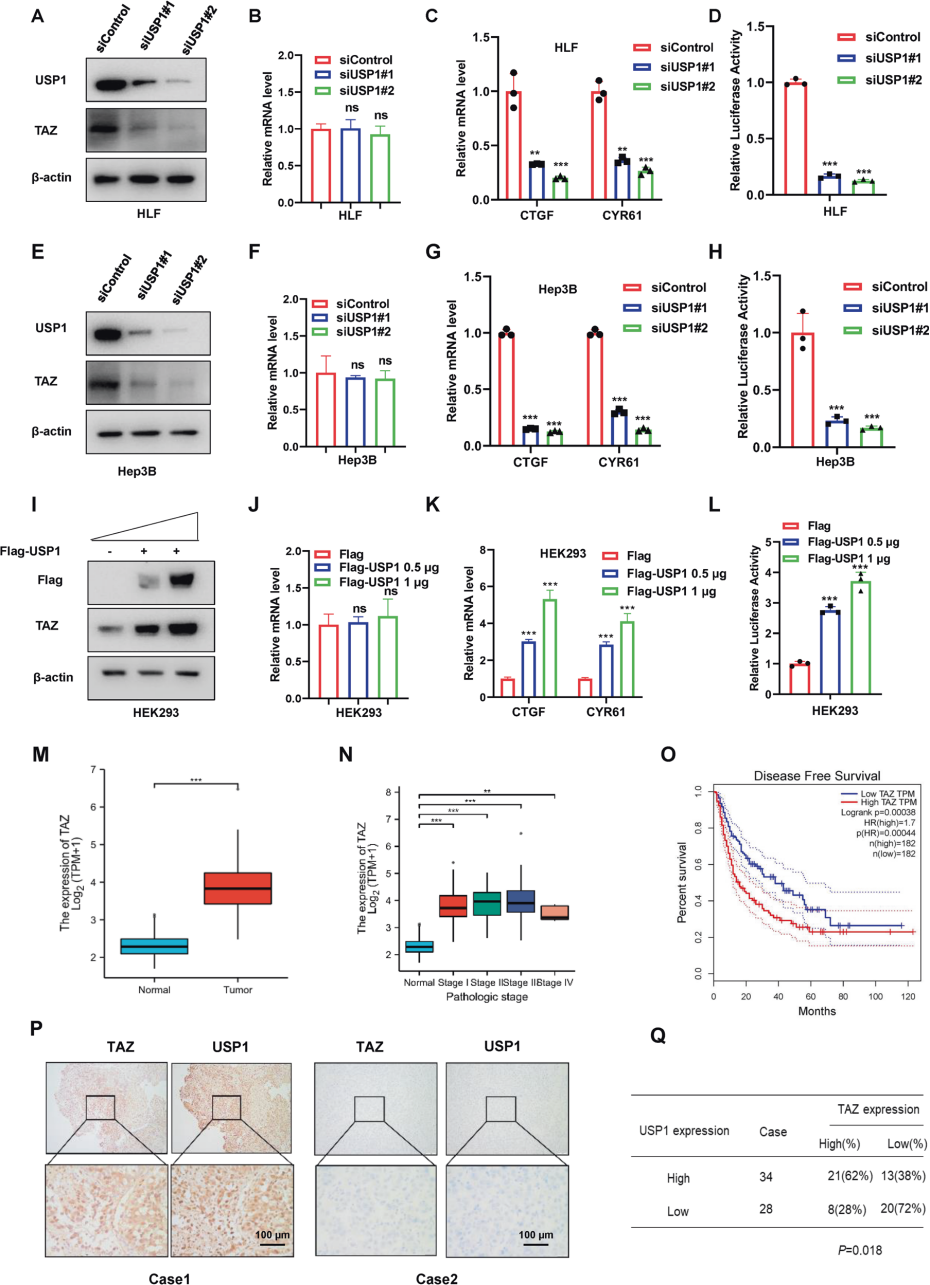
USP1 depletion inhibits Hippo/TAZ axis activity in HCC cells. **A**, **E**, **I** Western blot analysis of USP1 and TAZ expression in HLF, Hep3B, and HEK293 cells exposed as indicated. In this study, actin was employed as an internal reference. **B**, **F**, **J** RT–qPCR results of TAZ mRNA expression in HLF, Hep3B, and HEK293 cell lines exposed as indicated. **C**, **G**, **K** RT–qPCR results of CTGF and CYR61 mRNA expression in HLF, Hep3B, and HEK293 cell lines exposed as indicated. **D**, **H**, **L** In HLF, Hep3B, and HEK293 cells exposed as indicated, transcriptional activity of TEAD response elements was measured by a luciferase assay using a reporter containing tandem TEAD-binding sites. **M** In the TCGA database, the relative level of TAZ RNA in HCC tumor samples (*n* = 371) was compared to that in normal samples (*n* = 50) (https://www.genome.gov/). **N** In HCC tumors of various stages (Stag I, *n* = 168; Stag II, *n* = 84; Stag III, *n* = 82; Stag IV, *n* = 6), the relative RNA level of TAZ was compared with that in normal liver tissue (*n* = 50). Data were obtained from the TCGA database. (https://www.genome.gov/). **O** Comparing with lower TAZ expression (*n* = 275), higher TAZ expression (*n* = 90) was associated with poorer progression-free survival in HCC patients based on Kaplan‒Meier analysis in TCGA. *P* = 0.00044, log-rank test. **P**, **Q** Immunohistochemical (IHC) staining to evaluate USP1 and TAZ expression in HCC tissues. In Panels **A**–**L** the results are representative of three independent experiments. All the data are presented as the means ± SDs. **P < 0.01, ***P < 0.001 (Student’s *t* test).

In addition, we continued our study by depleting USP1 in HCC cell lines to confirm its biological link to Hippo signaling. To prevent off-target effects, we used two independent siRNAs targeting USP1. The immunoblotting data showed that depleting USP1 had no effect on the YAP protein level in HLF or Hep3B cells (data not shown). However, the TAZ protein level in HLF and Hep3B cells with USP1 depletion was dramatically decreased, as determined by Western blotting (Fig. 2A, E), while no significant change in mRNA expression was detected via qRT‒PCR (Fig. 2B, F). In addition, USP1 overexpression elevated the TAZ protein level (Figs. 2I and S1A, E). Gene expression analysis showed that USP1 depletion further decreased the expression of CTGF and CYR61, which are the most classical Hippo target genes, in HLF and Hep3B cells (Fig. 2C, G), while USP1 overexpression elevated the expression of these genes (Fig. 2K and S1C, G). The luciferase reporter assay showed that USP1 depletion in HLF and Hep3B cells inhibited TEAD response element activity (Fig. 2D, H), while USP1 overexpression enhanced TEAD response element activity (Figs. 2L and S1D, H). By performing chromatin immunoprecipitation experiments, we indicated that the silencing of USP1, could also restrained the ability of TEAD response element (CTGF) activity in figure S1I, J. According to reports, the TAZ level is elevated in some tumor tissues, such as breast and lung cancer tissues [25, 26]. After further inquiry into the expression of TAZ in hepatocellular carcinoma, we interestingly found that the mRNA level of TAZ was elevated in HCC tissues compared with normal liver tissues in the TCGA database (Fig. 2M). Moreover, TAZ expression was also elevated in each pathological stage of HCC compared with normal hepatocellular tissues (Fig. 2N). Further research in TCGA showed that high TAZ expression was related to poor survival in HCC patients (Fig. 2O). Moreover, from the TCGA dataset, we can observe that a strong positive correlation between USP1 and TAZ in liver cancer in Fig. S1K, which strengthened our conclusion that USP1 modulated Hippo target gene expression via TAZ. In addition, further analysis of USP1 and TAZ expression revealed a positive correlation between USP1 and TAZ protein expression in 62 samples of hepatocellular carcinoma collected from Qilu Hospital (*p* = 0.018; Fig. 2P, Q).

### USP1 depletion inhibits hepatocellular carcinoma progression in vivo and in vitro

**Fig. 3:**
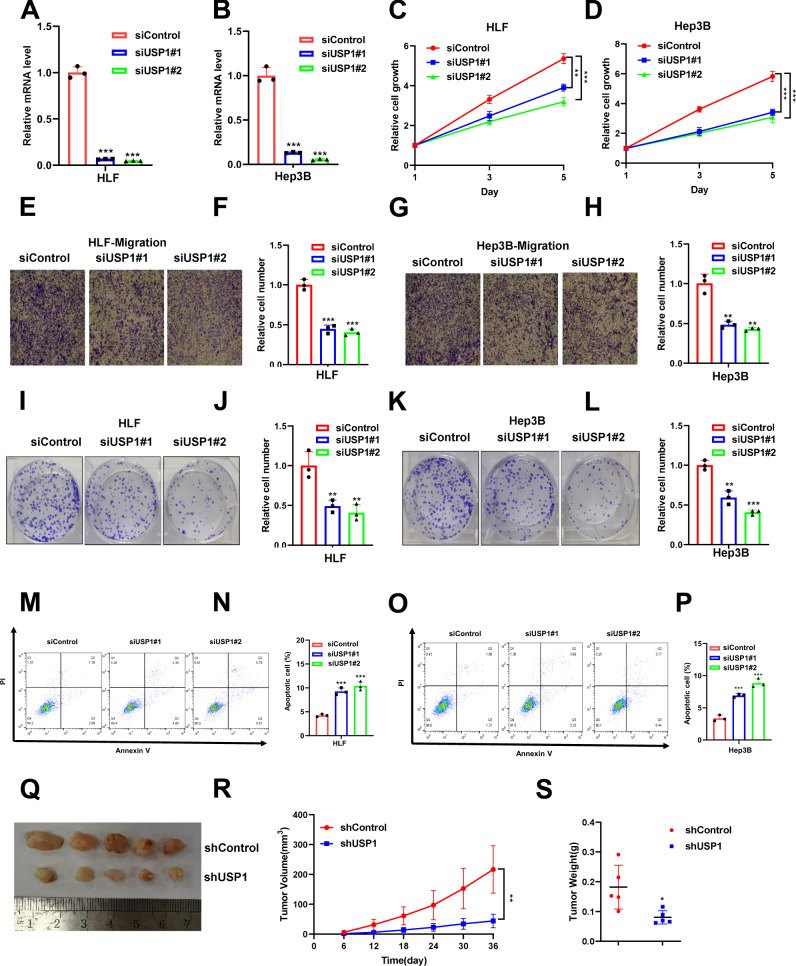
USP1 depletion inhibits hepatocellular carcinoma progression in vivo and in vitro. **A**, **B** RT‒PCR was performed to determine USP1 mRNA levels in HLF and Hep3B cells following USP1 siRNA treatment. **C**, **D** HLF and Hep3B cells transfected with siControl or siUSP1 were tested for viability using the CCK-8 assay at the indicated time points. **E**–**H** HLF and Hep3B cells were tested for their migration ability using Transwell assays. F and H show the quantitative analysis of the colony formation assay results. **I**–**L** Colony formation (left panel) of HLF and Hep3B cells transfected with scrambled siRNA or one of two independent USP1 siRNAs. **J** and **L** show the quantitative analysis of the colony formation assay results. **M**–**P** The percentage of apoptotic cells was determined by FACS analysis after HLF and Hep3B cells were treated with USP1 siRNA. PI and Annexin V staining were performed on the cells. **Q** A representative image of a tumor derived from a nude mouse injected with stably transfected shControl or shUSP1 HLF cells is shown. **R**, **S** The tumor volume (**R**) and weight (**S**) in nude mice subcutaneously inoculated with stably transfected shControl or shUSP1 HLF cells. Three independent experiments were conducted to obtain the results shown in Panels **A**–**P**. All the data are presented as the means ± SDs. **P < 0.01, ***P < 0.001 (Student’s *t* test).

We further examined the impact of USP1 on the hepatocellular carcinoma phenotype by depleting USP1 in HLF and Hep3B cells. We found that USP1 depletion was successfully achieved, as validated by qPCR. (Fig. 3A, B). The CCK8 assay showed that USP1 depletion sharply inhibited hepatocellular carcinoma cell proliferation (Fig. 3C, D). In addition, the Transwell assay indicated that USP1 depletion diminished the migration capacity of HLF and Hep3B cells (Fig. 3E–H). The colony formation assay confirmed that USP1 depletion decreased the colony formation capacity (Fig. 3I–L). In addition, we investigated the effect of USP1 on apoptosis, and propidium iodide (PI)/Annexin V staining revealed that USP1 depletion increased the proportions of apoptotic cells among HLF and Hep3B cells (Fig. 3M-1P). Moreover, a subcutaneous xenograft tumorigenesis model was established by randomly dividing nude mice into two groups and treating them with shControl or shUSP1 by subcutaneous injection. The xenograft mouse model used to study the effects of USP1 in vivo confirmed that silencing USP1 in HLF cells inhibited tumor growth in vivo (Fig. 3Q–S). We further validate the functional effects of USP1 depletion on HCC cells proliferation, migration and apoptosis by key regulators protein involved in the related pathways by western blotting. Our results showed that USP1 depletion significantly inhibited the protein level of Ki67 and N-cadherin in hepatocellular carcinoma cell. In addition, E-cadherin and Cleaved-casepase3 was promoted by USP1 depletion in Fig. S2A, B. In summary, according to all these data, USP1 plays a critical role in facilitating the progression of hepatocellular carcinomas.

### Hepatocellular carcinomas with TAZ overexpression display partial reversal of the antitumor effects of USP1 depletion

**Fig. 4:**
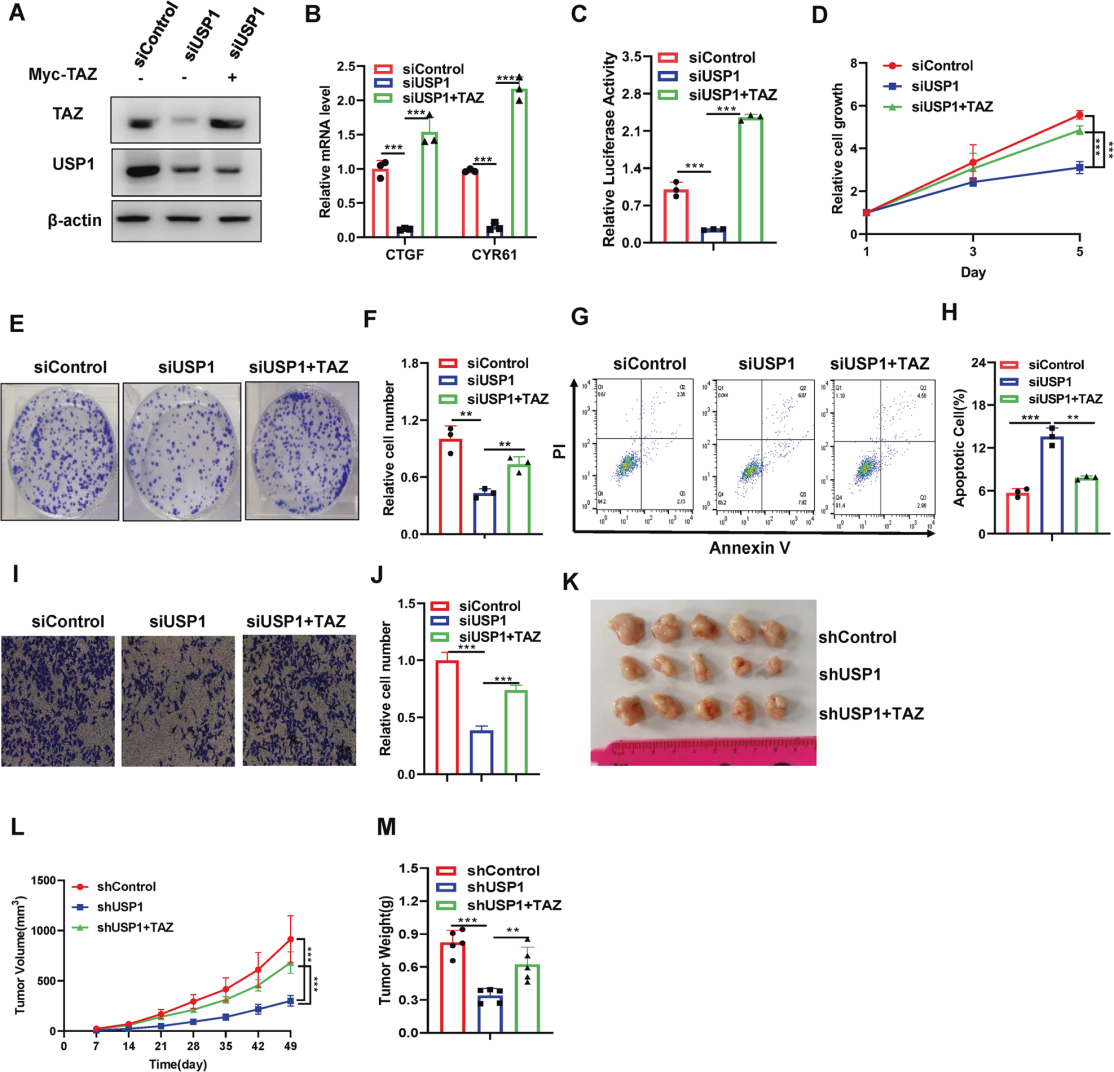
Hepatocellular carcinomas with TAZ overexpression display partial reversal of the antitumor effects of USP1 depletion. **A** Western blot analysis of TAZ and USP1 expression in HLF cells treated with siControl or siUSP1 for 24 h, then transfected with Myc or Myc-TAZ for another 24 h. In this study, β-Actin was used as the internal reference. **B** Results of RT‒qPCR to measure CTGF and CYR61 mRNA levels in HLF cells treated as indicated. **C** TEAD response element transcriptional activity was measured via luciferase reporter assays with tandem TEAD-binding sites in HLF cells treated as indicated. **D** At the indicated time points, a CCK-8 assay was performed to determine the viability of HLF cells treated as indicated. **E**, **F** Colony formation (left panel) of HLF cells treated as indicated. F shows the quantitative analysis of the colony formation assay results. **G**, **H** FACS analysis (left panel) was performed on HLF cells treated as indicated. **I**, **J** Transwell assays was performed on HLF cells transfected treated as indicated. **K** As indicated, images of tumors derived from nude mice injected with shControl, shUSP1- or shUSP1+TAZ-transfected HLF cells are shown. **L**, **M** Tumor volume (**L**) and weight (**M**) in nude mice injected with stably transfected shControl, shUSP1, or shUSP1+TAZ HLF cells. In Panels **A**–**J**, the results are representative of three independent experiments. In Panels **K**–**M**, the results are representative of five independent experiments. The data are presented as the means ± SDs. **P < 0.01, ***P < 0.001 (Student’s *t* test).

To inquire whether TAZ is involved in USP1-mediated proliferation, migration, colony formation and apoptosis in hepatocellular carcinoma cells, we carried out several rescue experiments. HLF cells were transfected with siControl or siUSP1 for 24 h, and then transfected with Myc or Myc-TAZ. The immunoblot data showed that USP1 knockdown decreased the protein level of TAZ, which could be reversed by further TAZ overexpression (Fig. 4A). The qPCR data indicated that the expression of Hippo target genes, which was downregulated by USP1 depletion, was rescued by further TAZ overexpression (Fig. 4B). The luciferase reporter assay showed that USP1 depletion in HLF cells inhibited TEAD response element activity and that this phenotype was reversed by further TAZ overexpression (Fig. 4C). The CCK8 assay indicated that USP1 depletion significantly inhibited hepatocellular carcinoma cell growth, which was partially reversed by further TAZ overexpression (Fig. 4D), and these results were confirmed by the colony formation assays (Fig. 4E, F). In addition, USP1 depletion promoted apoptosis in HLF cells, while further TAZ overexpression partially reduced the percentage of apoptotic cells (Fig. 4G, H). Furthermore, Transwell assay indicated that USP1 depletion significantly inhibited hepatocellular carcinoma cell migration capacity, which was partially reversed by further TAZ overexpression (Fig. 4I, J). Moreover, the results in the xenograft mouse model indicated that shUSP1 inhibited HCC tumor growth from HLF cells, while further TAZ overexpression at least partially reversed the growth inhibition caused by USP1 depletion (Fig. 4K–M). These results indicate that TAZ partially accounts for the antitumor effect of USP1 depletion. We also indicate that TAZ partially accounts for the tumor effect of USP1 overexpression by constructing Flag, Flag-USP1 and Flag-USP1 + shTAZ stably HLF cell line (Fig. S3A–M).

### USP1 interacts with TAZ and modulates TAZ stability in hepatocellular carcinoma cells

**Fig. 5:**
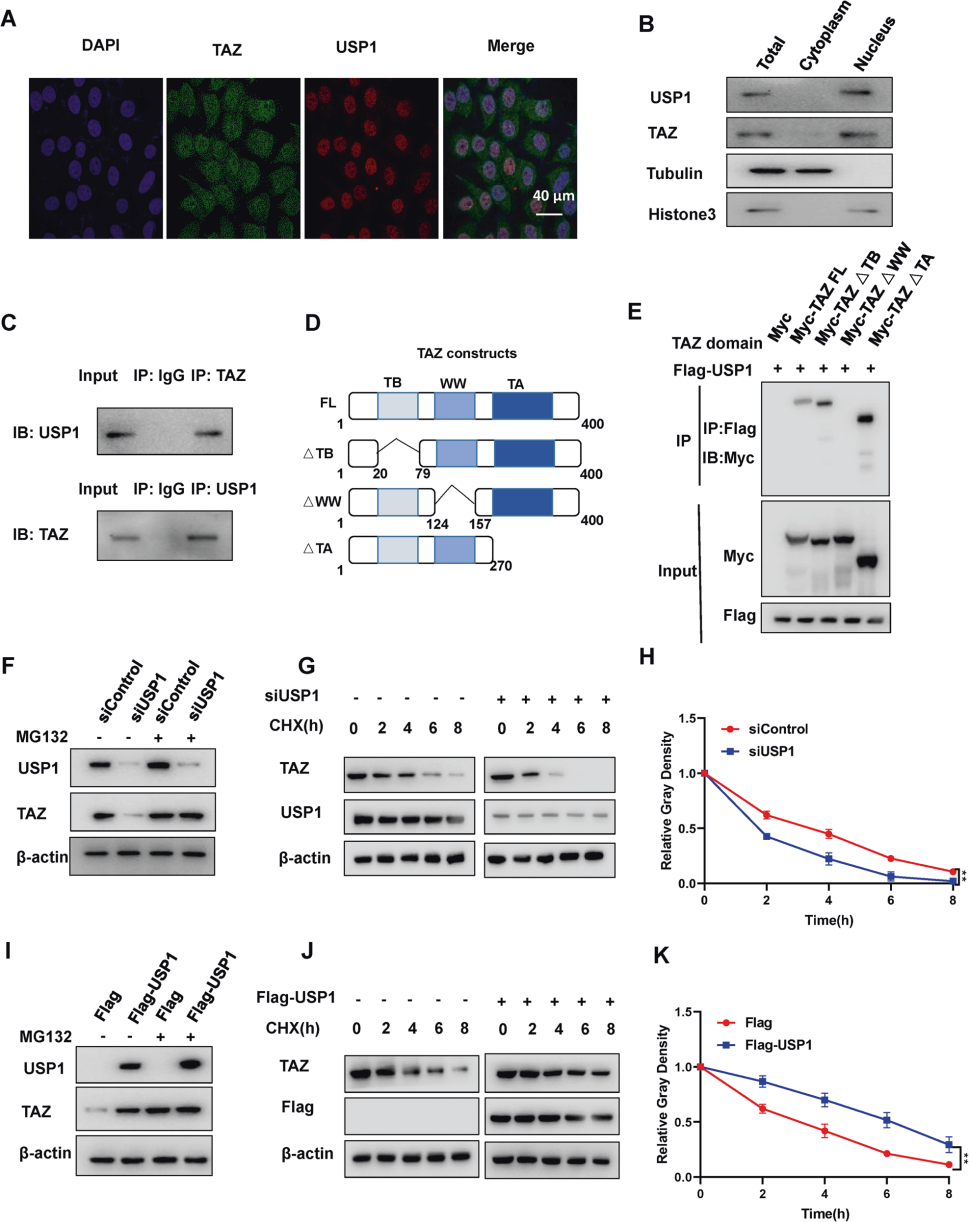
In hepatocellular carcinoma cells, USP1 interacts with TAZ and modulates its stability. **A** Immunofluorescence imaging of TAZ (green), USP1 (red) and DAPI (blue) in HLF cells, scale bar 40 µm. **B** Western blot analysis of USP1 and TAZ protein localization in HLF cells. In this study, the cytoplasmic and nuclear fractions were separated using a subcellular protein fractionation kit (Thermo Scientific 78840). The control cytoplasmic and nuclear fractions were analyzed using antibodies specific for tubulin and histone3, respectively. **C** USP1 and TAZ proteins were found to be associated in HLF cells by a coimmunoprecipitation (Co-IP) assay. **D** The wild-type and truncated TAZ constructs are shown in a schematic diagram. **E** An immunoblot demonstrating the interaction between USP1 and WT TAZ or truncated TAZ, as assessed by Co-IP with USP1 (with an anti-Flag antibody). **F**, **I** Western blotting were used to measure TAZ and USP1 protein levels in HLF and HEK293 cells treated as indicated. MG132 (10 μM) was applied to the cells for 8 h before western blot analysis was performed. **G**, **H** USP1 depletion decreased the TAZ protein half-life in HLF cells. Cells were treated with 100 μmol/L CHX for the indicated time periods before being collected for western blot analysis. **H** Quantitative analysis of the half-life of the TAZ protein. **J**, **K** USP1 prolonged the TAZ protein half-life in HEK293 cells. Cells were treated with 100 μmol/L CHX for the indicated time periods before being collected for western blot analysis. **K** Quantitative analysis of the half-life of the TAZ protein. All the results are representative of 3 independent experiments. The data are presented as the means ± SDs. **P < 0.01 (Student’s *t* test).

In addition, we examined the localization of USP1 and TAZ in hepatocellular carcinoma cells. Immunostaining revealed that both proteins were mainly found in the nucleus (Fig. 5A). This finding was further confirmed by a nuclear/cytoplasmic fractionation assay (Fig. 5B). USP1 interacted with TAZ in HLF cells, as shown by endogenous immunoprecipitation (Fig. 5C). The TAZ protein is composed of three domains: the TB domain (TEAD binding), the WW domain, and the TA domain (Fig. 5D). By generating the corresponding deletion constructs and investigating the associated domain via an IP assay, we were able to demonstrate that TAZ interacts with USP1 through the WW domain (Fig. 5E). USP1 depletion decreased the TAZ protein level in HLF cells, and MG132 treatment reversed the USP1 depletion-induced reduction in the TAZ protein level in HLF cells (Fig. 5F). Consistent with this finding, USP1 overexpression in HEK293 cells appeared to increase the TAZ protein level, and this effect was minimized by MG132 treatment (Fig. 5I). According to these data, we proposed that USP1 modulates the protein level of TAZ via the proteasomal degradation system. In addition, we further evaluated TAZ protein stability in HLF cells with USP1 silencing and found that USP1 depletion greatly decreased TAZ protein stability (Fig. 5G, H). This finding was further confirmed in HEK293 cells via USP1 overexpression (Fig. 5J, K).

### USP1 regulates TAZ protein stability by inhibiting K11-linked polyubiquitination of TAZ

**Fig. 6:**
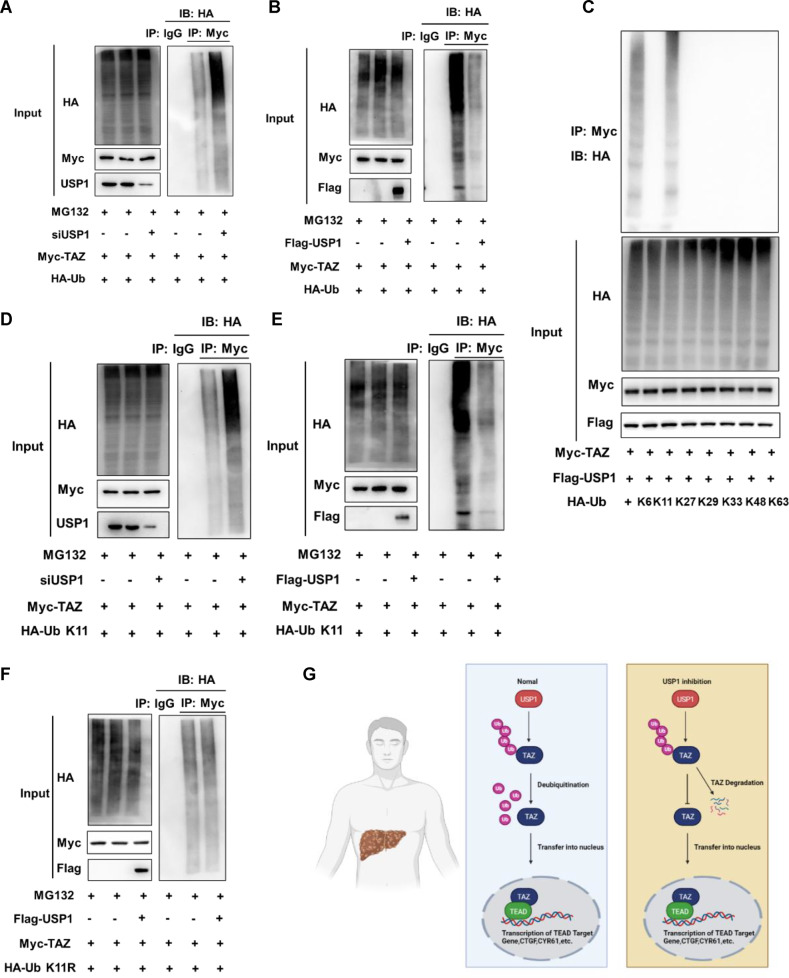
USP1 regulates TAZ protein stability by inhibiting K11-linked polyubiquitination of TAZ. **A** Western blot analysis of polyubiquitinated TAZ was performed after coimmunoprecipitation (Co-IP) in HLF cells treated as indicated. **B**, **C** Western blot analysis of polyubiquitinated TAZ was performed after coimmunoprecipitation in HEK293 cells treated as indicated. **D**, **E** Western blot analysis of K11 linkage-specific polyubiquitinated TAZ was performed after coimmunoprecipitation in HLF or HEK293 cells treated as indicated. **F** Western blot analysis of K11R linkage-specific polyubiquitinated TAZ was performed after coimmunoprecipitation in HEK293 cells treated as indicated. **G** The hypothetical mechanistic model of USP1-regulated Hippo signaling in HCC progression. The USP1 associates with TAZ and decreases TAZ protein degradation via K11-linked polyubiquitination of TAZ, which increases Hippo/TAZ axis activation and enhances malignant progression in HCC cells. All the results are representative of three independent experiments.

USP1 is a member of the USP family of deubiquitinating enzymes; thus, we then evaluated whether USP1 can regulate the polyubiquitination level of the TAZ protein. We demonstrated that USP1 depletion increased the total polyubiquitination level of TAZ in HLF cells via endogenous proteins (Fig. 6A). A polyubiquitination assay in HEK293 cells revealed that USP1 overexpression decreased the total polyubiquitination level of TAZ (Fig. 6B). Eight different types of polyubiquitination linkages have been reported, seven of which are linked to seven conserved lysine residues, namely, K6 (Lys6), K11 (Lys11), K27 (Lys27), K29 (Lys29), K33 (Lys33), K48 (Lys48), and K63 (Lys63) [27]. Subsequently, we further explored which subtypes of ubiquitin chains are involved in TAZ modification and are regulated by USP1. Our research showed that USP1 depletion specifically increased K11-linked ubiquitination (Fig. 6C). In addition, these results were validated via ubiquitin-based immunoprecipitation assays in HLF and HEK293 cells, as indicated (Fig. 6D–F). Collectively, these data indicate that USP1 regulates TAZ protein stability by reducing the K11-linked polyubiquitination of TAZ.

## Discussion

In our research, by screening a DUB siRNA library, we discovered a novel member of the deubiquitinase family, USP1. This enzyme is a critical endogenous modulator of the Hippo/TAZ axis that promotes hepatocellular carcinoma. USP1 expression was elevated in human hepatocellular carcinoma and associated with poor survival in hepatocellular carcinoma patients. In addition, when we further analyzed the correlation of USP1 expression with that of classical downstream genes of the Hippo signaling pathway in the TCGA database with *p* < 0.001 as the threshold, we found that USP1 had significant positive correlations with 44 genes, including AMOTL2, ASAP1, CCN1 (CYR61), and CCN2 (CTGF), which exactly validated our DUB siRNA library screening results. Moreover, these conclusions were confirmed by RNA sequencing analysis in HLF cells with USP1 depletion. In addition, USP1 inhibition caused cell growth inhibition and decreased the cell migration capacity. Further results revealed that USP1 was required for Hippo/TAZ axis activity and HCC progression. Furthermore, USP1 interacted with the WW domain of TAZ, which subsequently enhanced TAZ stability by inhibiting its K11-linked polyubiquitination. Our research revealed a novel mechanism linking USP1 and TAZ in Hippo pathway regulation and one possible therapeutic target for HCC (Fig. 6G). Based on these findings, we propose that pharmaceutically targeting USP1 function or inhibiting USP1 expression may be effective in suppressing Hippo/TAZ-driven hepatocellular carcinoma.

The Hippo signaling pathway is thought to function as a major tumor suppressor pathway by inhibiting hepatocyte proliferation, survival, and invasion and hepatocellular carcinogenesis. HCC samples showed increased expression of the Hippo pathway effectors YAP and TAZ [7], which is associated with poor overall survival; for example, TAZ plays an essential role in c-MYC-induced hepatocarcinogenesis [28]. Furthermore, hepatocyte plasticity and differentiation-specific hepatocarcinogenesis are determined by Yap-Sox9 signaling [29]. In addition, under hypoxic stress, YAP can associate with the HIF-1α protein and maintain its stability to enhance glycolysis in hepatocellular carcinoma [21]. Therefore, targeting the Hippo signaling effectors YAP/TAZ could be a promising therapeutic direction for hepatocellular carcinoma.

The ubiquitin‒proteasome system (UPS) is one of the most important pathways in regulating protein degradation [30]. Ubiquitination is a reversible process whereby ubiquitin molecules bind to target proteins for proteasomal degradation through an enzymatic cascade of ubiquitin-activating enzymes (E1), ubiquitin-binding enzymes (E2), and ubiquitin ligases (E3) via ATP. Ubiquitin signaling is regulated by deubiquitinating enzymes, which cleave the ubiquitin chain or remove ubiquitin moieties from modified substrates, thereby reversing the ubiquitination of the target proteins. Deubiquitinating enzymes are extensively implicated in the regulation of cellular functions, including proteasome/lysosome-associated protein degradation, cell cycle regulation, DNA repair, kinase activation, and gene expression [31], which play a vital role in tumor-based diseases and can be used as potential diagnostic and therapeutic targets for cancer.

Previous research has indicated that ubiquitination-mediated regulation plays an important role in the activation and inactivation of the Hippo signaling pathway [7]. According to Jianing Tang et al., USP26 and TAZ form a complex that decreases TAZ ubiquitination levels and regulates anaplastic thyroid cancer progression [32]. Furthermore, our previous studies verified that OTUB1 and DUB1 promote gastric cancer cell progression and invasion by positively regulating Hippo signaling [22, 33]. OTUB1 was found to interact with YAP and subsequently inhibit YAP degradation. In contrast, USP36 repressed Hippo signaling by regulating TAZ protein ubiquitination levels in gastric cancer. However, deubiquitinating enzymes that regulate Hippo signaling pathway activity have not been systematically explored in hepatocellular carcinoma.

To systematically explore the critical DUBs in the modulation of Hippo signaling activity, we employed one DUB siRNA library (Dharmacon Company, Cat: G104705) containing 100 siRNAs targeting each DUB to screen for important DUBs. We identified USP1 as a novel effector of TAZ stability and hepatocellular carcinoma progression. This finding identified not only novel endogenous modulators of Hippo signaling but also promising therapeutic targets in the Hippo/TAZ pathway in hepatocellular carcinoma. Moreover, USP1 is reportedly a promising target for regulating the progression of hepatocellular carcinoma. According to Yuning Liao et al., the stability of the RPS16 protein is regulated by USP1 and is critical to hepatocellular carcinoma cell growth and metastasis [34]. Consistent with their findings, our TCGA data analysis revealed that USP1 expression was elevated in HCC and associated with poor survival in HCC patients. In addition, this conclusion was confirmed by RNA sequencing analysis in HLF cells (GSE218366). The volcano plot indicated that USP1 depletion significantly facilitated the expression of classical Hippo pathway target genes, including CTGF and CYR61. In addition, Hippo signaling was largely affected by USP1 depletion, according to KEGG pathway analysis, and these results were confirmed by GSEA. Moreover, the expression of USP1 was positively associated with TAZ protein expression, according to our analysis of HCC clinical samples. Considering these results collectively, we propose that USP1 could be a positive regulator of the Hippo/TAZ axis in human hepatocellular carcinoma.

There are, however, still many unknowns regarding the specific roles of USP1 in HCC progression and its underlying mechanisms. According to Mussell et al. [23], USP1 and TAZ form a complex that affects TAZ ubiquitination and is associated with breast cancer metastasis. Furthermore, Yuan et al. [24] also reported that osteosarcoma progression is suppressed by USP1 inhibition via destabilization of TAZ. Consistent with their findings, dual-luciferase reporter assays, immunofluorescence assays, cycloheximide assays and polyubiquitination assays, etc., revealed that USP1 was required for Hippo/TAZ axis activity. USP1 interacted with the WW domain of TAZ, which subsequently enhanced TAZ stability by inhibiting K11-linked polyubiquitination of TAZ. In our study, we proposed a regulatory link between USP1 and the Hippo/TAZ pathway and a potential target for HCC treatment.

## Conclusions

We identified USP1 as an oncogene in hepatocellular carcinoma. We revealed that USP1 expression was promoted in hepatocellular carcinoma and associated with poor survival. USP1 interacted with the TAZ protein and suppressed its K11-linked polyubiquitination in hepatocellular carcinoma cells. As USP1 modulates Hippo signaling, modulating its activity or gene expression level is a promising strategy for the treatment of hepatocellular carcinoma.

## Supplementary information

USP1-TAZ original WB data

Supplemental figure legends

aj-checklist

supplementary figure 1

supplementary figure 2

supplementary figure 3

## Data Availability

The RNA sequencing data have been deposited in the Gene Expression Omnibus (GEO) database (GEO: GSE218366). Full and uncropped western blots can be found in the Supplementary Material.
