# Supplementary material for: USP1 modulates hepatocellular carcinoma progression via the Hippo/TAZ axis
Source: Cell Death Dis. 2023 Apr 12;14(4):264. doi: 10.1038/s41419-023-05777-1 (PMC10090121; doi:10.1038/s41419-023-05777-1)

Figure 2

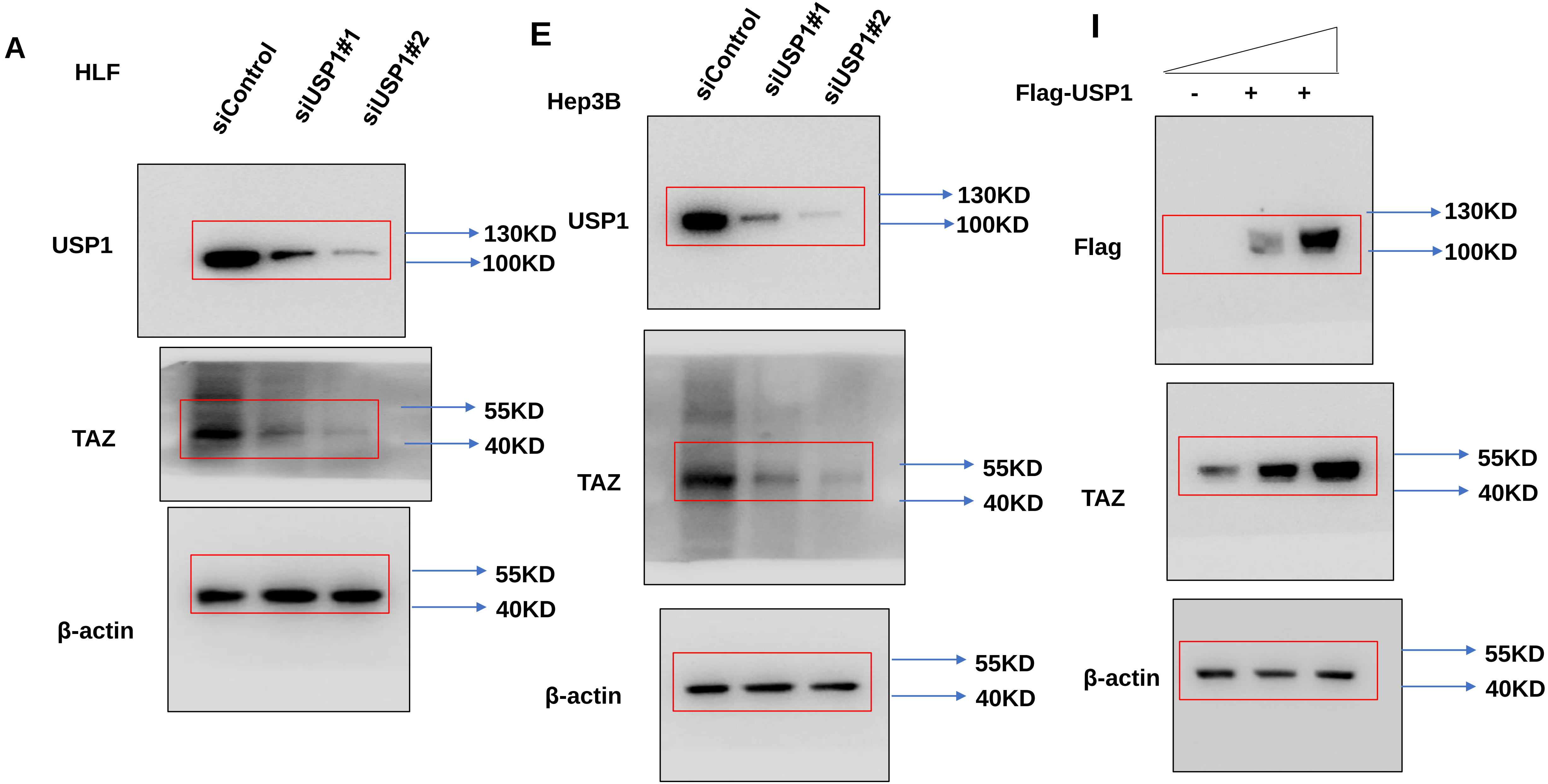

A
HLF
siControl
siUSP1#1
siUSP1#2
USP1
130KD
100KD
TAZ
55KD
40KD
β-actin
55KD
40KD
E
Hep3B
siControl
siUSP1#1
siUSP1#2
USP1
130KD
100KD
TAZ
55KD
40KD
β-actin
55KD
40KD
I
Flag-USP1
-
+
+
Flag
130KD
100KD
TAZ
55KD
40KD
β-actin
55KD
40KD

Figure 4

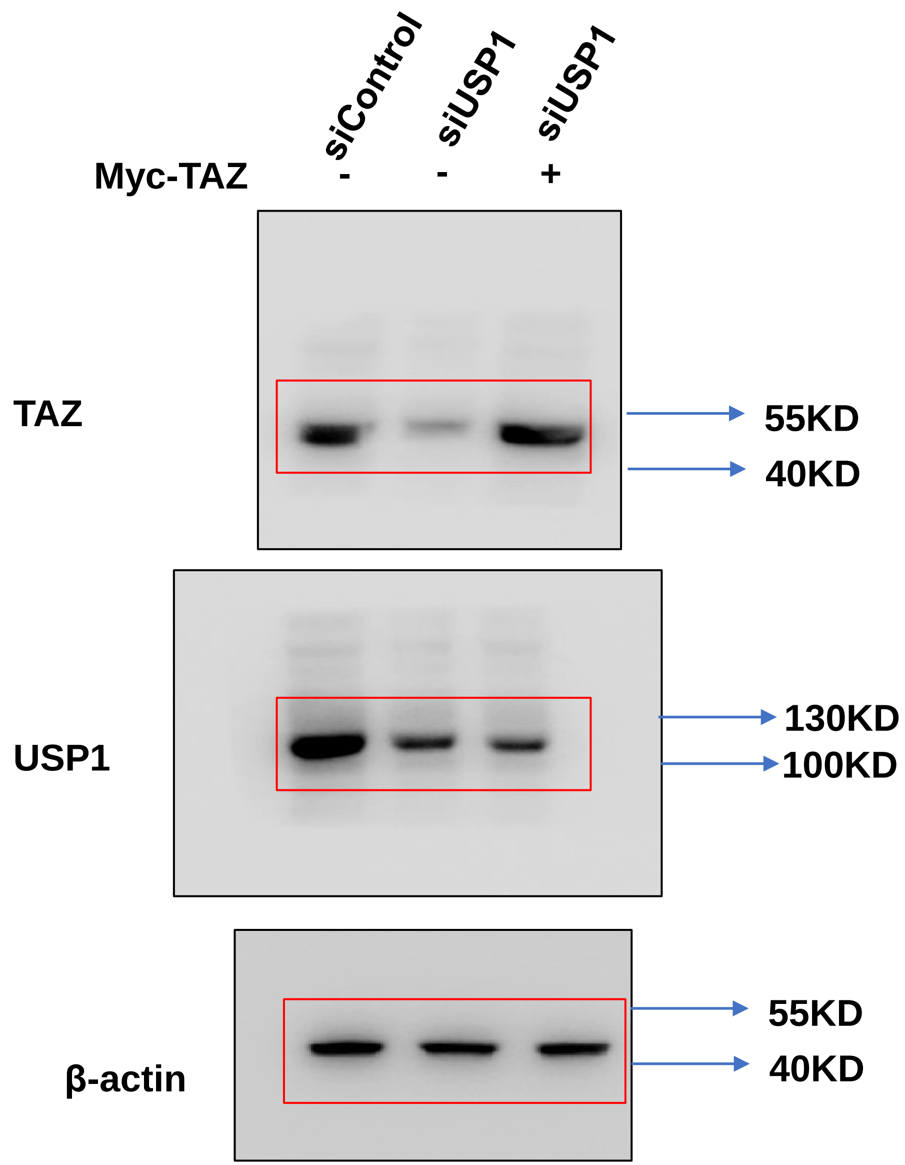
siControl
siUSP1
siUSP1
Myc-TAZ
-
-
+
TAZ
55KD
40KD
USP1
130KD
100KD
β-actin
55KD
40KD

Figure 5

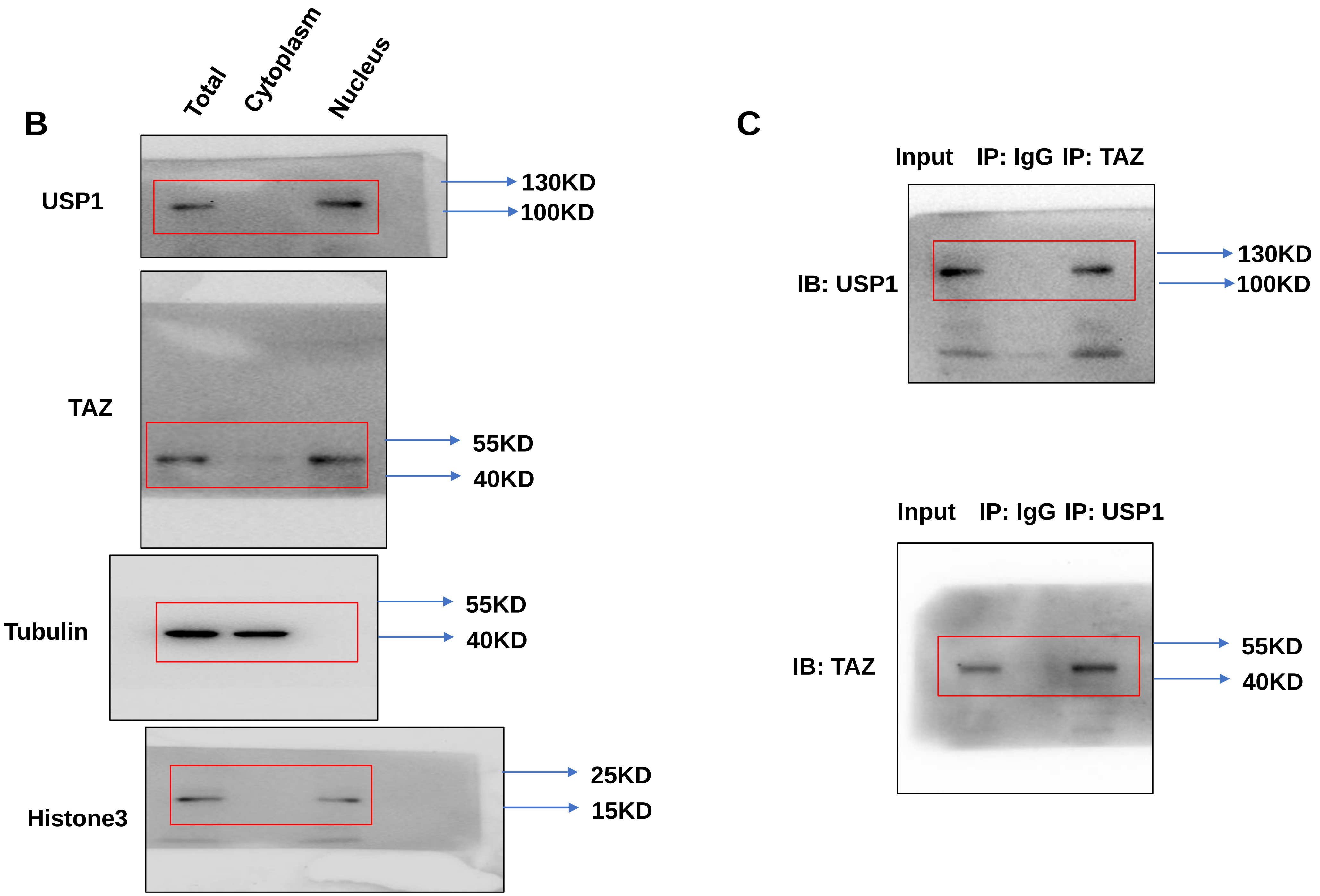
B
Total
Cytoplasm
Nucleus
USP1
130KD
100KD
TAZ
55KD
40KD
Tubulin
55KD
40KD
Histone3
25KD
15KD
C
Input
IP: IgG
IP: TAZ
IB: USP1
130KD
100KD
Input
IP: IgG
IP: USP1
IB: TAZ
55KD
40KD

Figure 5 E

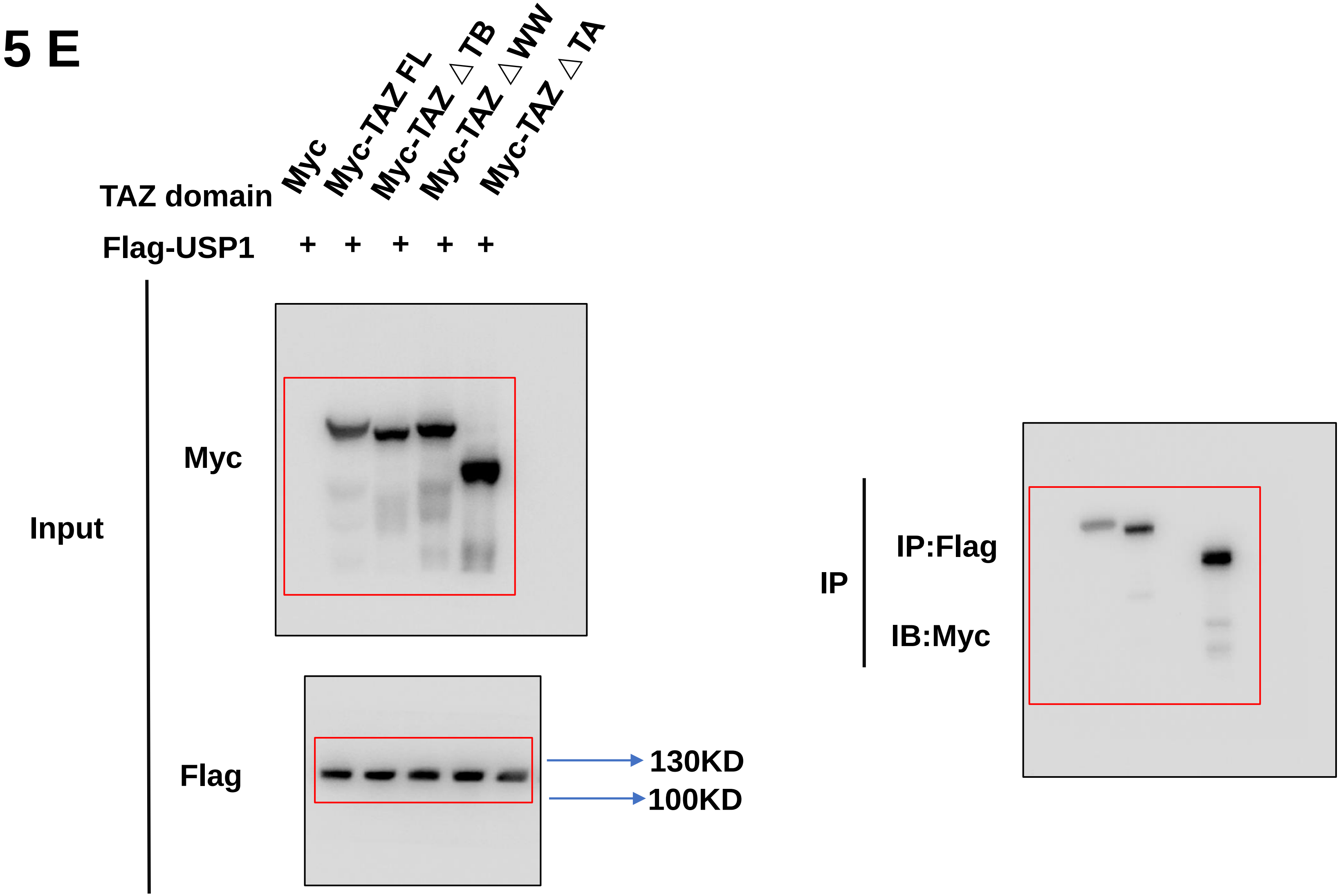

TAZ domain
Myc
Myc-TAZ FL
Myc-TAZ △TB
Myc-TAZ △WW
Myc-TAZ △TA
Flag-USP1
+ + + + +
Input
Myc
Flag
130KD
100KD
IP
IP:Flag
IB:Myc

Figure 5 F

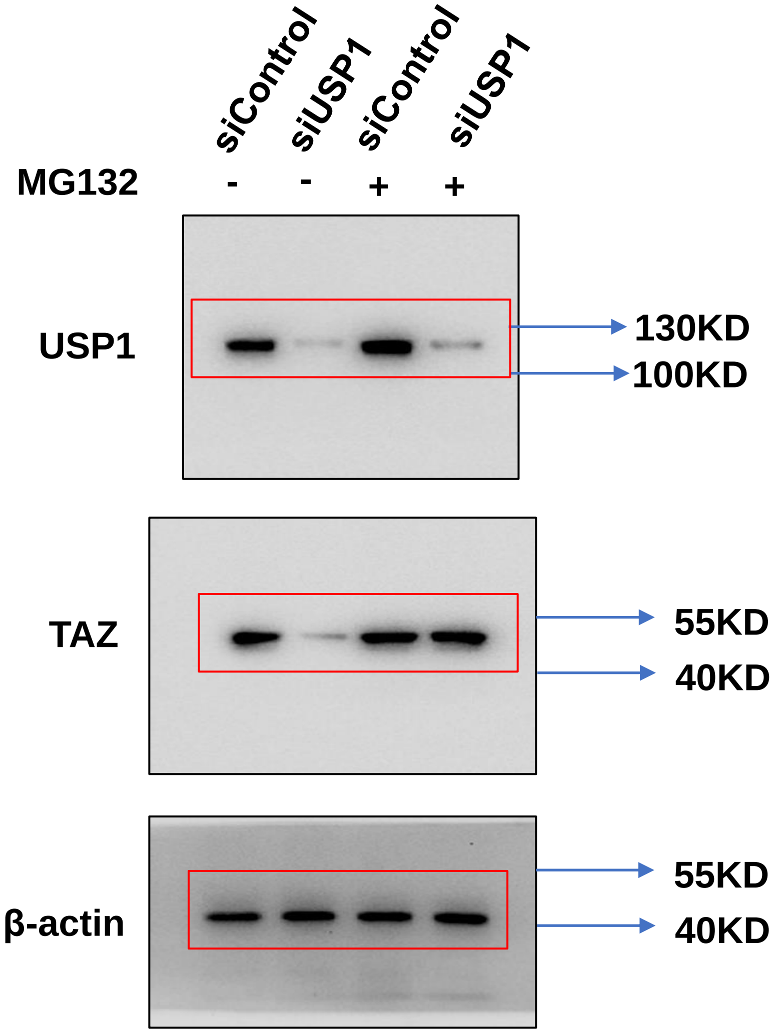
siControl
siUSP1
siControl
siUSP1
MG132
-
-
+
+
USP1
130KD
100KD
TAZ
55KD
40KD
β-actin
55KD
40KD

Figure 5 G

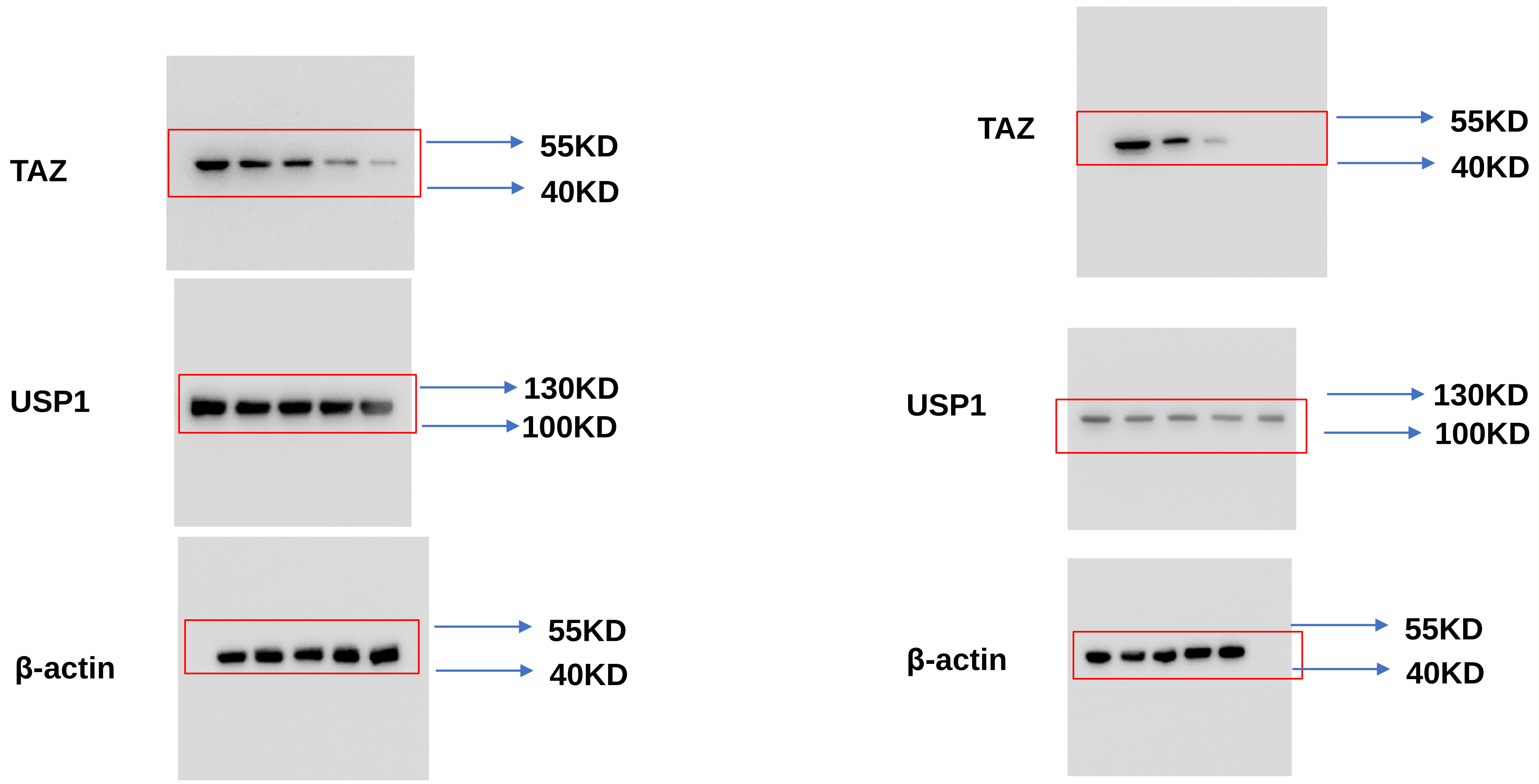
TAZ
55KD
40KD
USP1
130KD
100KD
β-actin
55KD
40KD
TAZ
55KD
40KD
USP1
130KD
100KD
β-actin
55KD
40KD

Figure 5 I

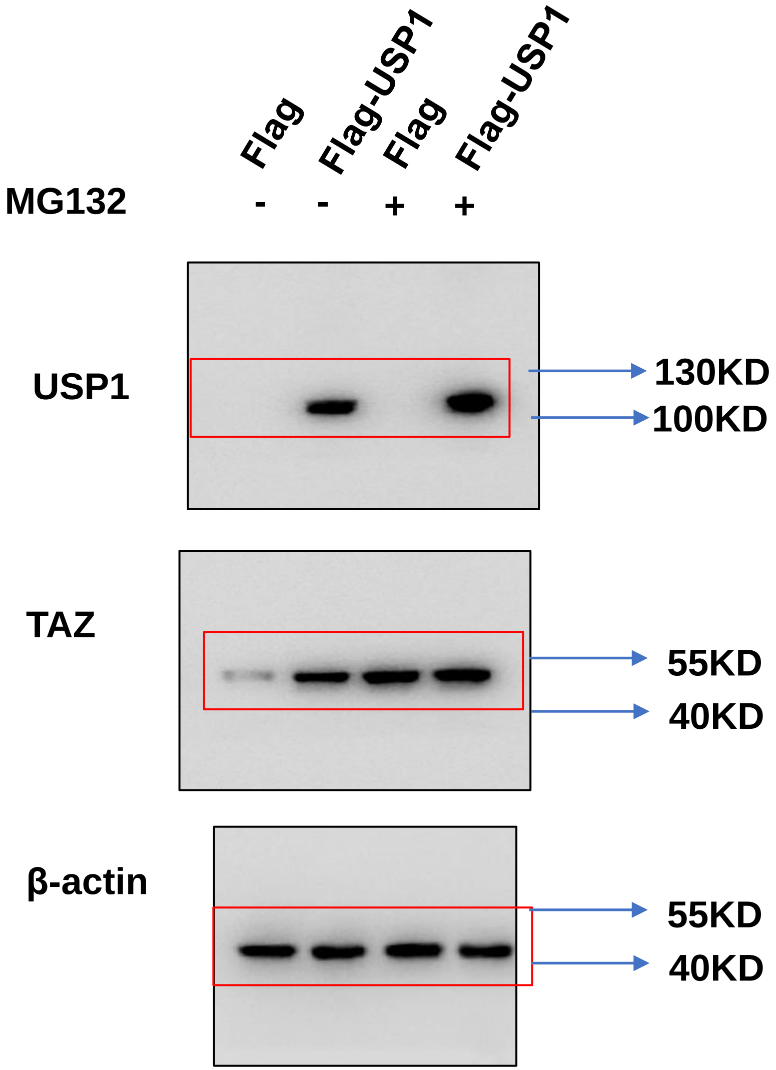

Flag
Flag-USP1
Flag
Flag-USP1
MG132
-
-
+
+
USP1
130KD
100KD
TAZ
55KD
40KD
β-actin
55KD
40KD

Figure 5 J

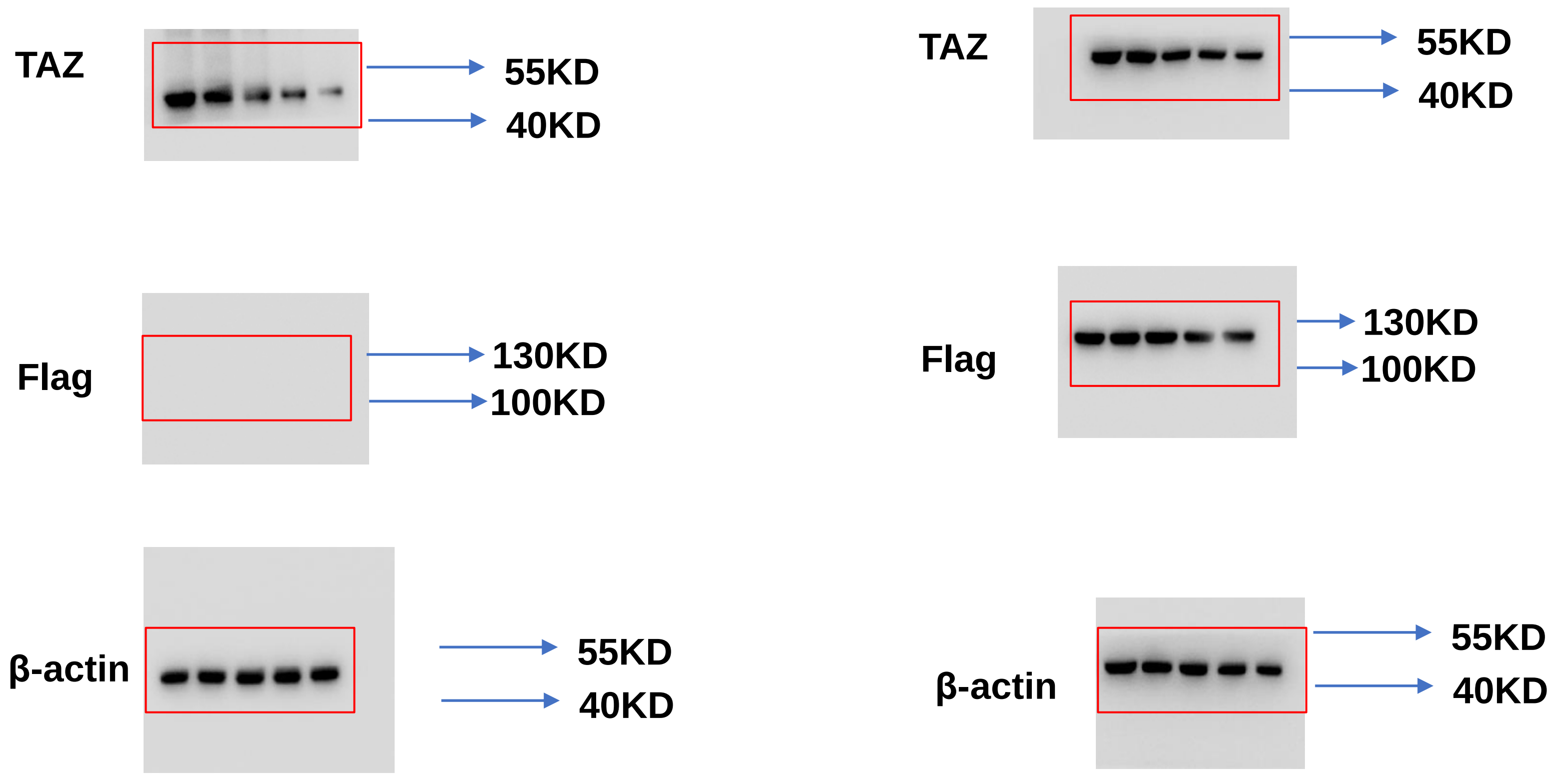
TAZ
55KD
40KD
Flag
130KD
100KD
β-actin
55KD
40KD
TAZ
55KD
40KD
Flag
130KD
100KD
β-actin
55KD
40KD

Figure 6 A

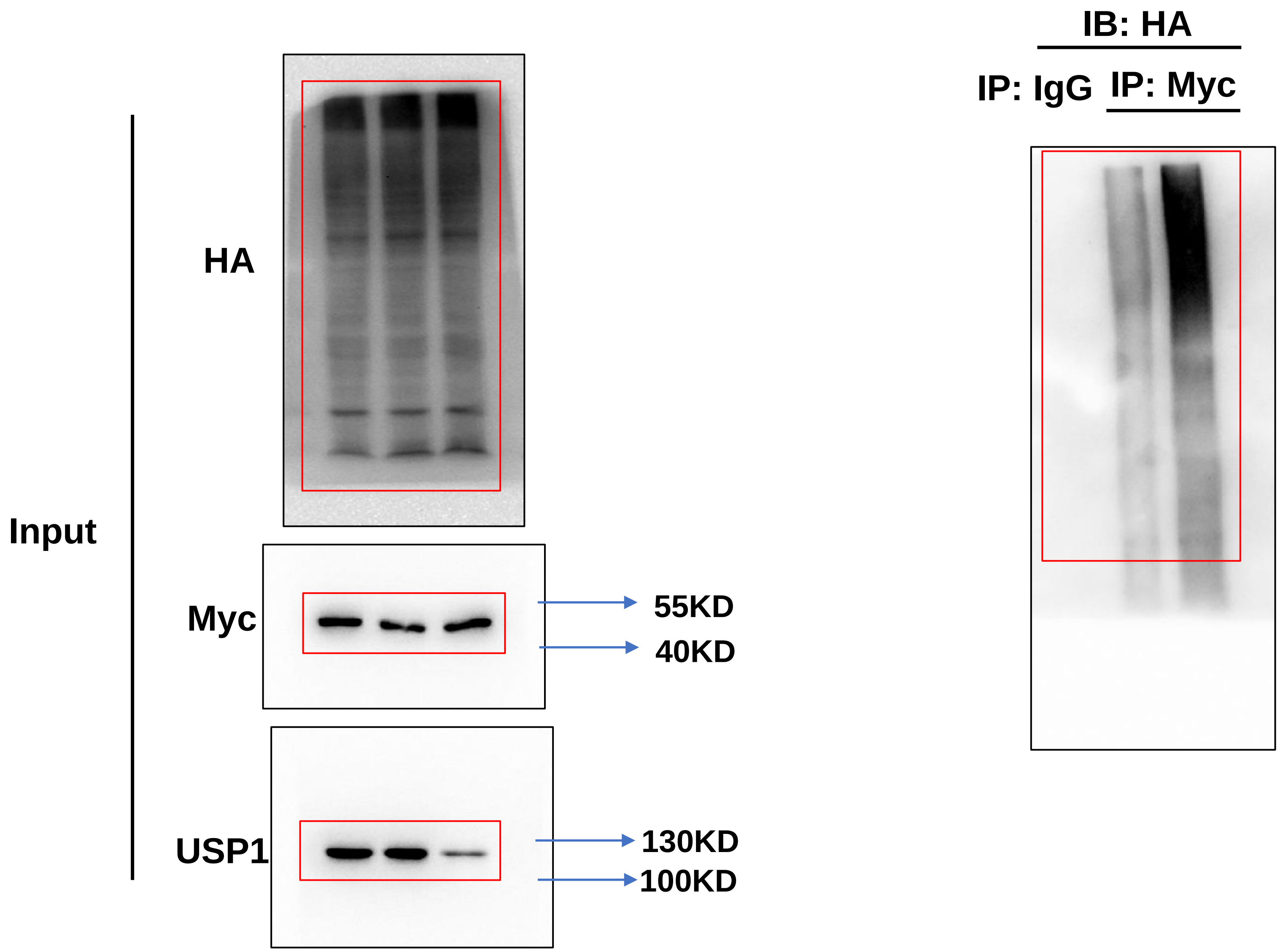

Figure 6 B

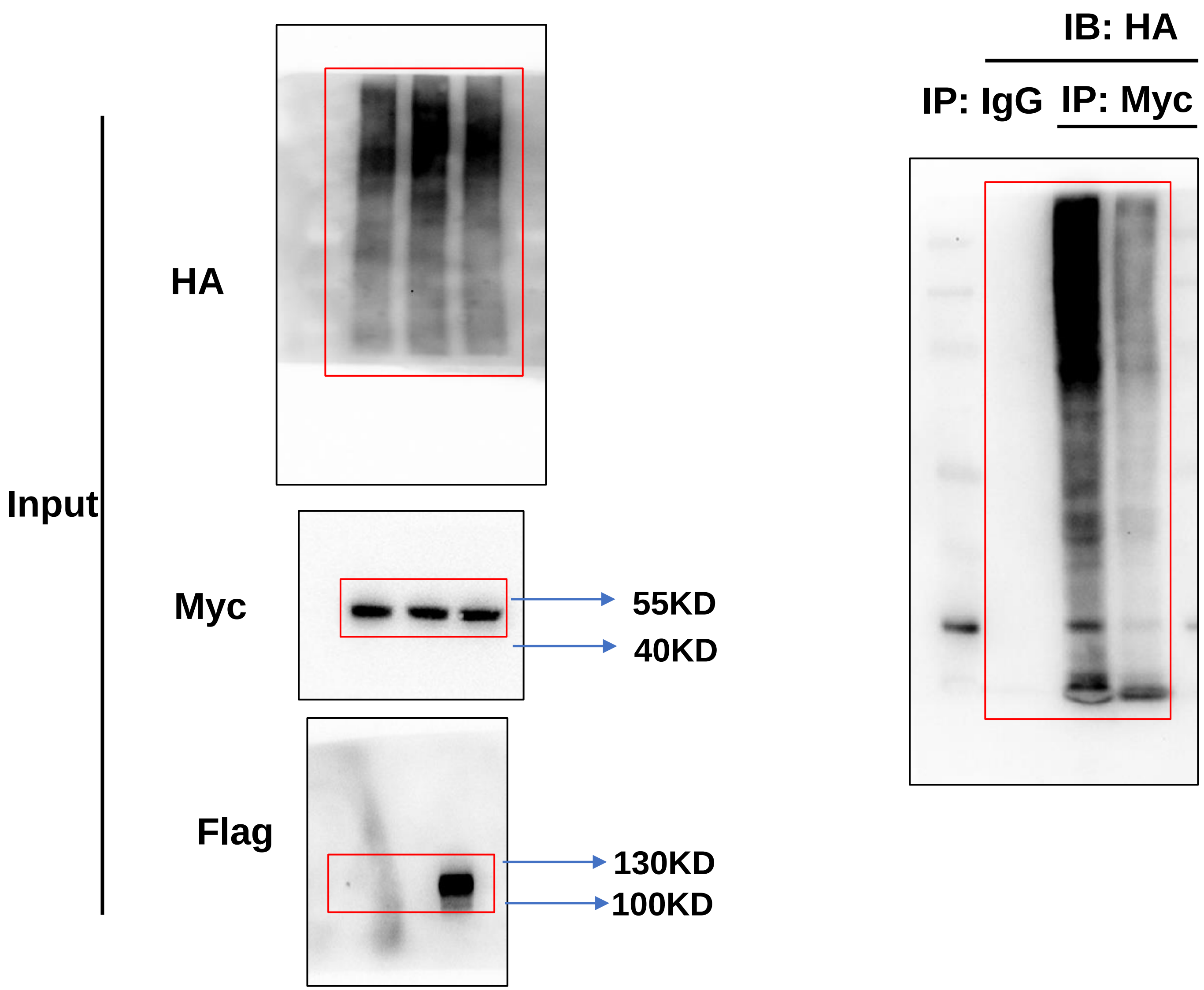

Figure 6 C

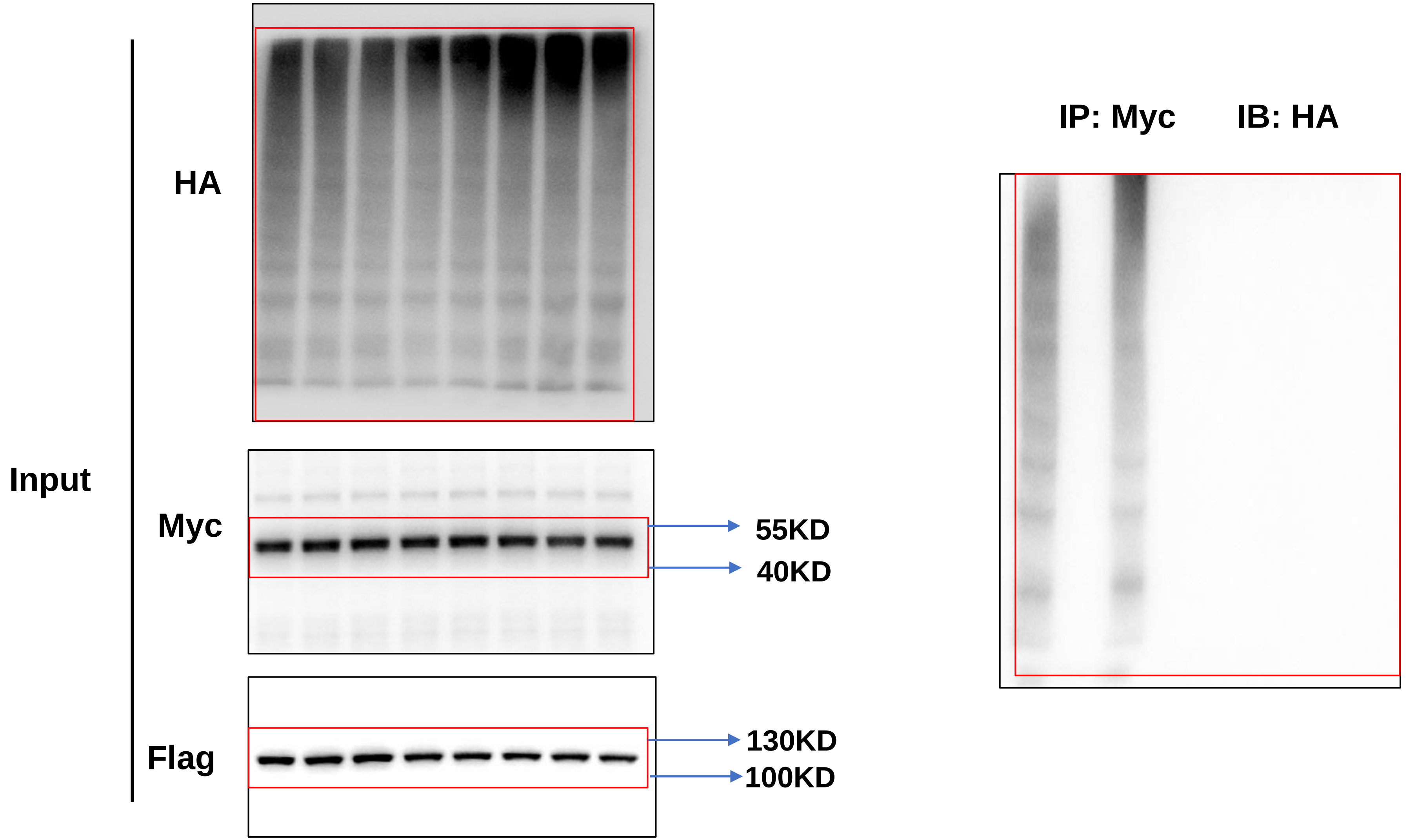

Figure 6 D

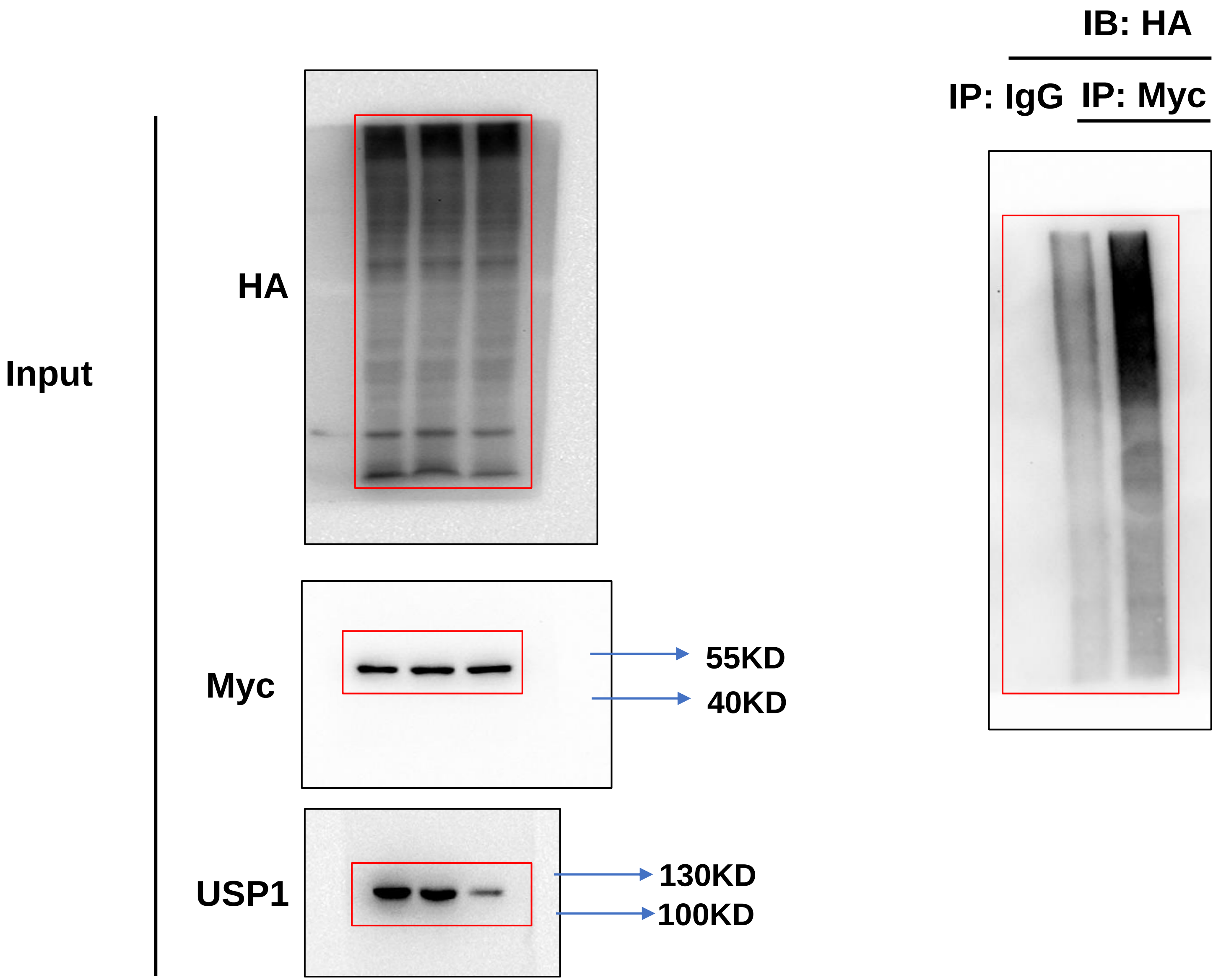

Figure 6 E

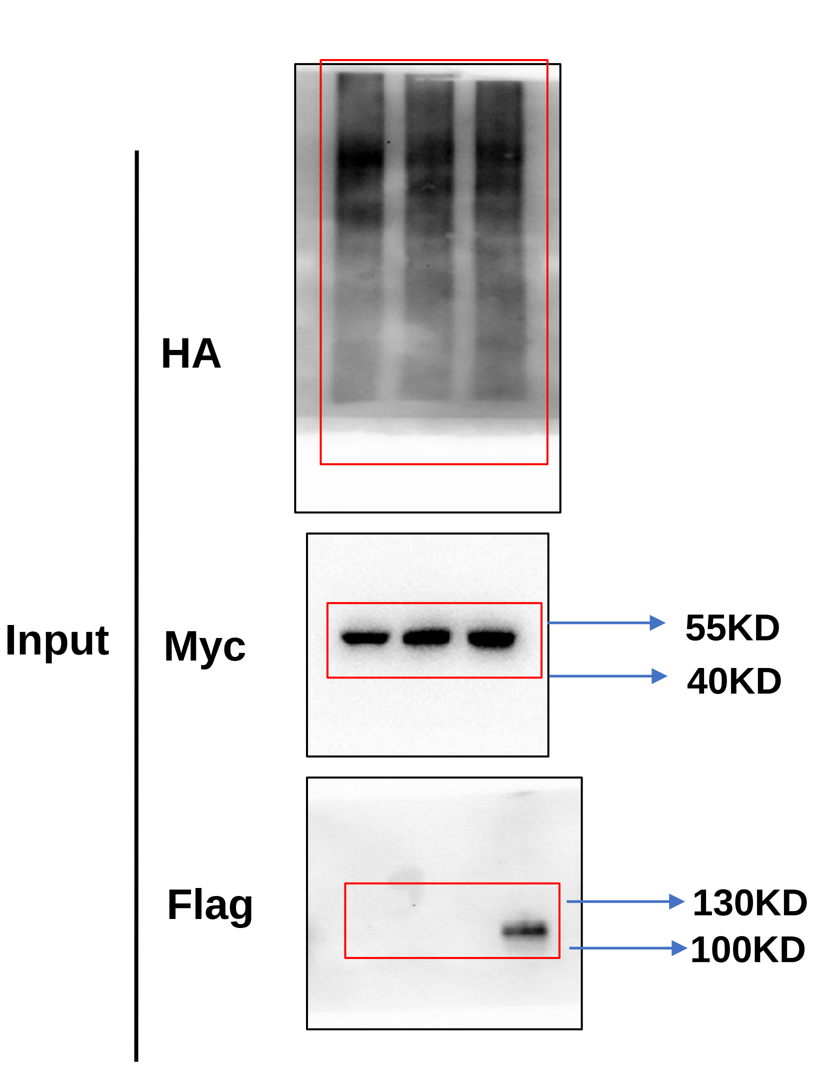

Figure 6 F

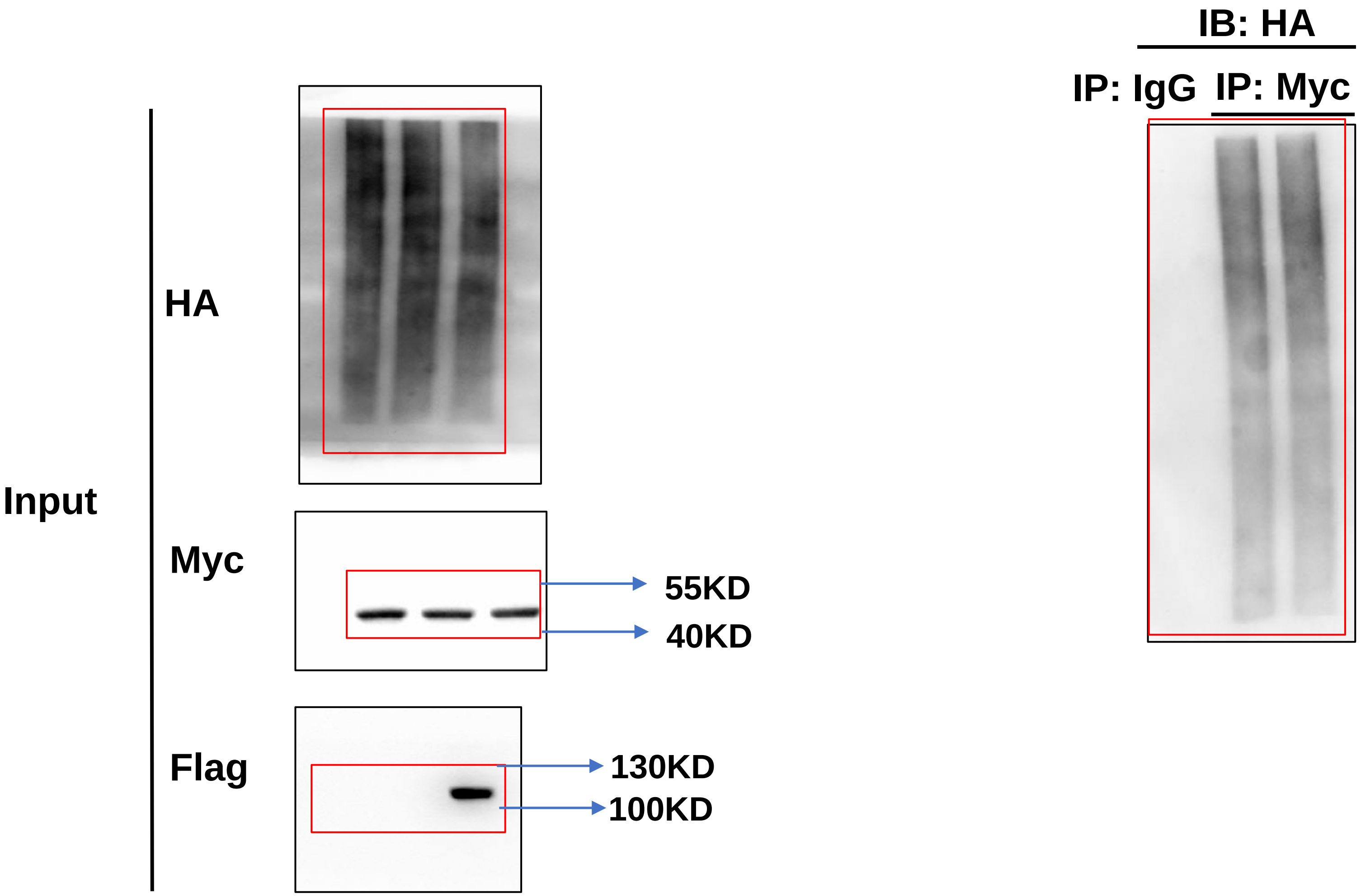

Supplementary figure 1

A

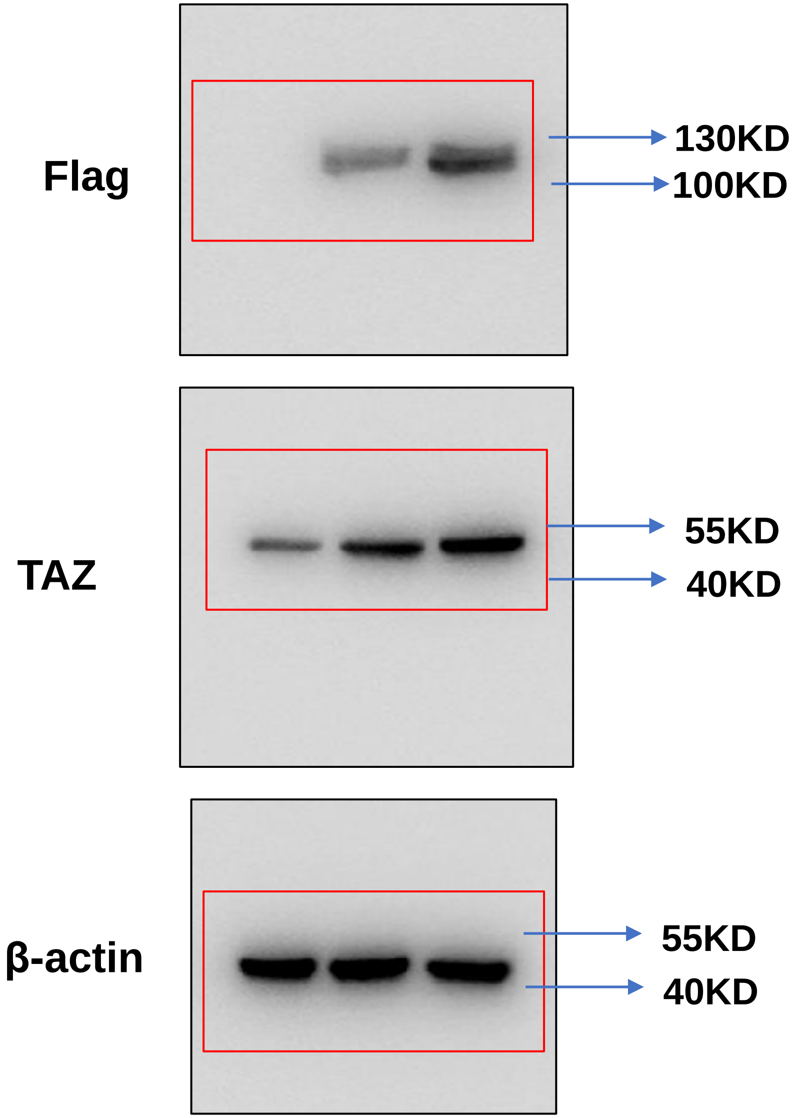

E

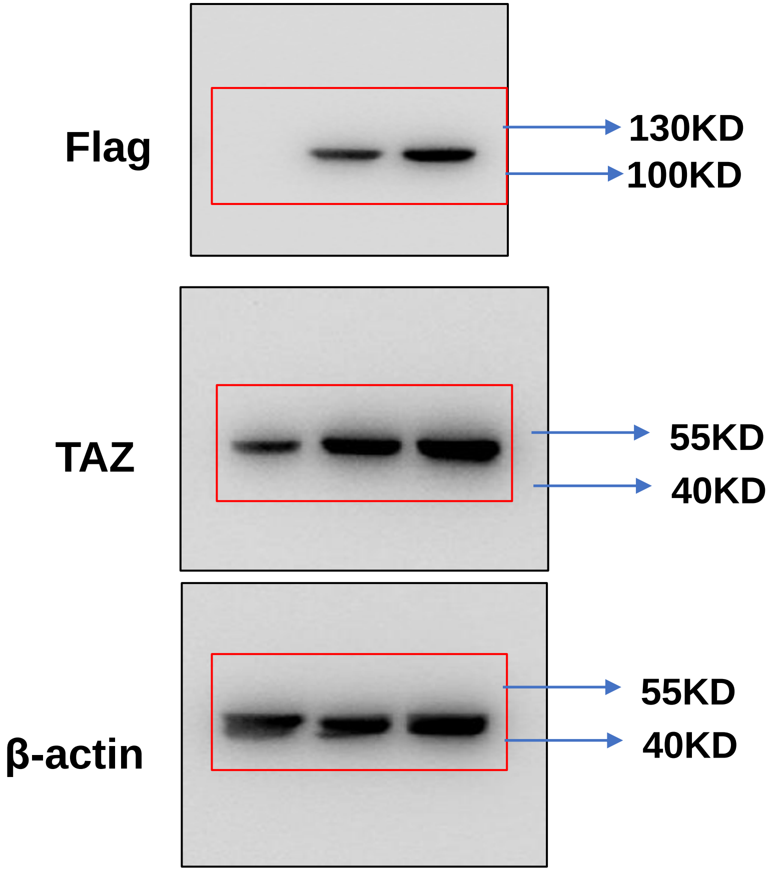

Supplementary figure 2

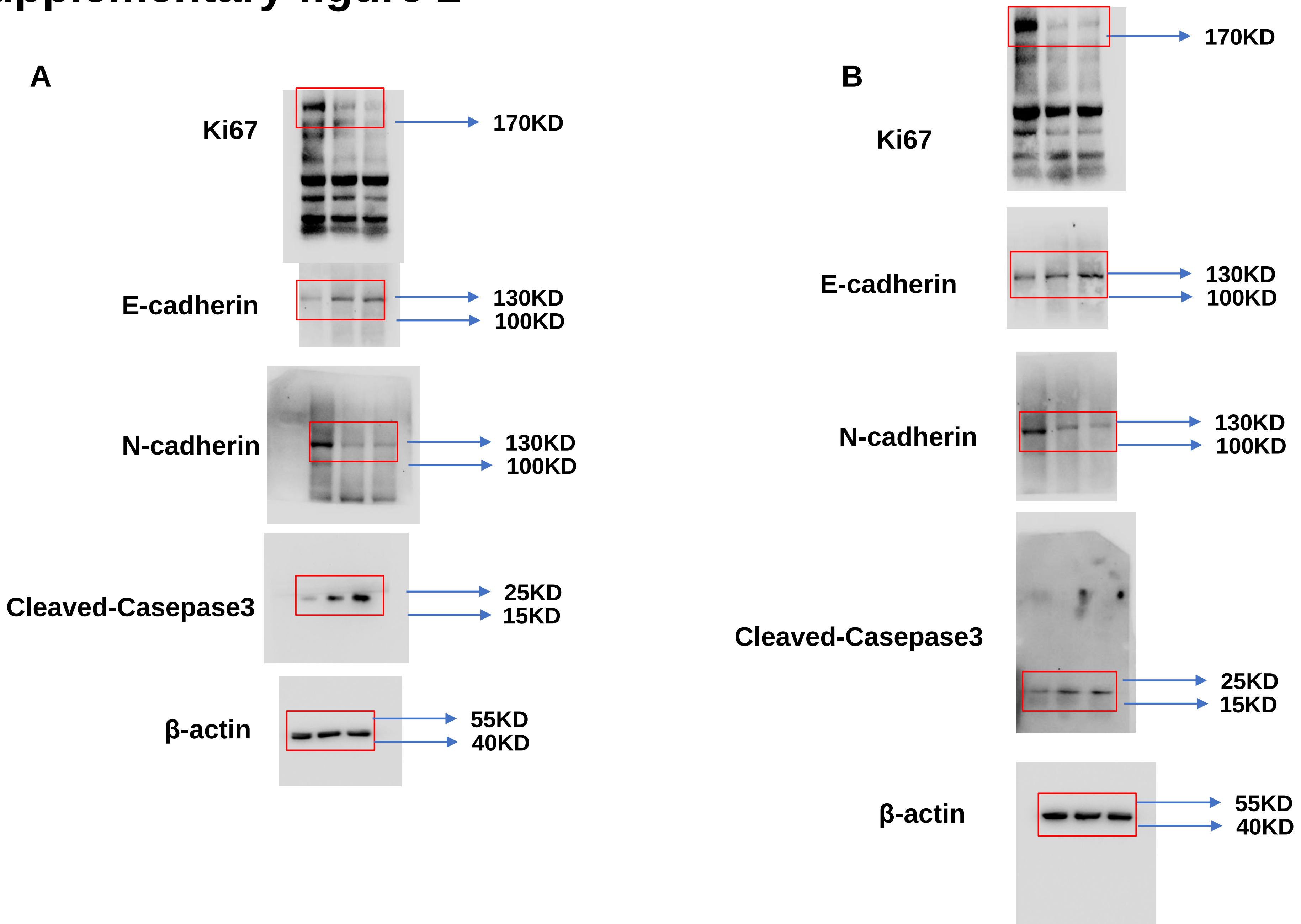

Supplementary figure 3

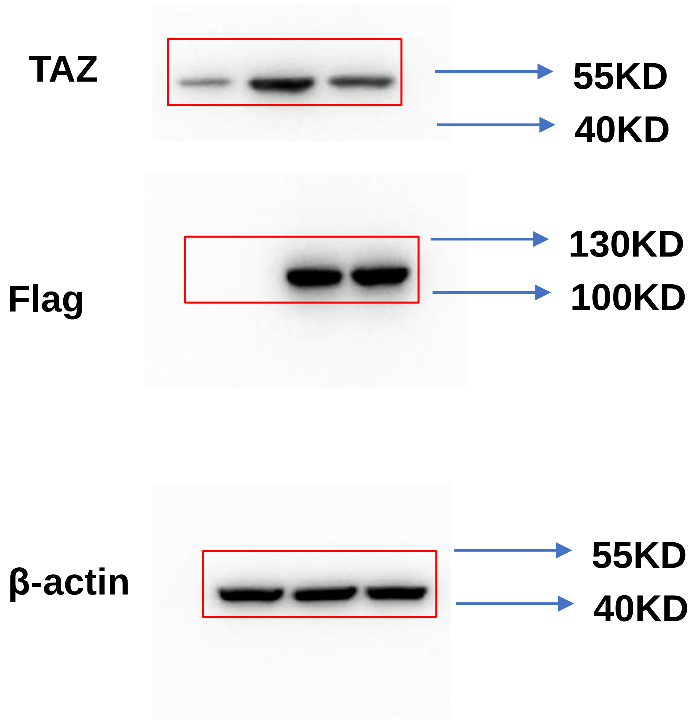

Supplement: Supplementary file 1 — USP1-TAZ original WB data [file 41419_2023_5777_MOESM1_ESM.pdf]
