# Supplementary material for: USP1 modulates hepatocellular carcinoma progression via the Hippo/TAZ axis
Source: Cell Death Dis. 2023 Apr 12;14(4):264. doi: 10.1038/s41419-023-05777-1 (PMC10090121; doi:10.1038/s41419-023-05777-1)
Supplement: Supplementary file 2 — Supplemental figure legends [file 41419_2023_5777_MOESM2_ESM.docx]

**Supplementary Figure 1**

**USP1 overexpression promotes Hippo/TAZ axis activity in HCC cells**

(A, E) Western blot analysis of USP1 and TAZ expression in HLF and Hep3B cells exposed as indicated. In this study, actin was employed as an internal reference.

(B, F) RT–qPCR results of TAZ mRNA expression in HLF and Hep3B cell lines exposed as indicated.

(C, G) RT–qPCR results of CTGF and CYR61 mRNA expression in HLF and Hep3B cell lines exposed as indicated.

(D, H) In HLF and Hep3B cells exposed as indicated, transcriptional activity of TEAD response elements was measured by a luciferase assay using a reporter containing tandem TEAD-binding sites.

(I, J) A ChIP assay was performed with anti-IgG or anti-TAZ antibody using HLF and Hep3B cells and CTGF mRNA levels were analyzed by quantitative RT-PCR.

(K) The correlation analysis of USP1 and TAZ in HCC (n=371) from TCGA database.

In Panels A-J, the results are representative of three independent experiments. The data are presented as the means ± SDs. ***P*<0.01, ****P*<0.001 (Student’s t test).

**Supplementary Figure 2**

(A-B) Western blot analysis of Ki67, N-cadherin, E-cadherin and Cleaved-casepase3 expression in HLF and Hep3B cells exposed as indicated. In this study, actin was employed as an internal reference.

**Supplementary Figure 3**

**Hepatocellular carcinomas with TAZ depletion display partial reversal of the tumor effects of USP1 overexpression.**

(A) Western blot analysis of TAZ and USP1 expression in Flag, Flag-USP1 and Flag-USP1+shTAZ stably HIF cell lines. β-Actin was used as the internal reference.

(B) Results of RT‒qPCR to measure CTGF and CYR61 mRNA levels in Flag, Flag-USP1 and Flag-USP1+shTAZ stably HIF cell lines.

(C) TEAD response element transcriptional activity was measured via luciferase reporter assays with tandem TEAD-binding sites in indicated cell lines.

(D) At the indicated time points, a CCK-8 assay was performed to determine the viability of indicated cell lines treated as indicated.

(E-F) Colony formation (left panel) of HLF cells treated as indicated. F shows the quantitative analysis of the colony formation assay results.

(G-H) FACS analysis (left panel) was performed on HLF cells treated as indicated.

(I-J) Transwell assays was performed on HLF cells transfected treated as indicated.

(K) As indicated, images of tumors derived from nude mice injected with Flag, Flag-USP1 or Flag-USP1+shTAZ-transfected HLF cells are shown.

(L-M) Tumor volume (L) and weight (M) in nude mice injected with stably transfected with Flag, Flag-USP1 or Flag-USP1+shTAZ-transfected HLF cells.

In Panels A-J, the results are representative of three independent experiments. In Panels K-M, the results are representative of six independent experiments. The data are presented as the means ± SDs. ***P*<0.01, ****P*<0.001 (Student’s t test).
