# Supplementary figures and images for: USP1 modulates hepatocellular carcinoma progression via the Hippo/TAZ axis
Source: Cell Death Dis. 2023 Apr 12;14(4):264. doi: 10.1038/s41419-023-05777-1 (PMC10090121; doi:10.1038/s41419-023-05777-1)

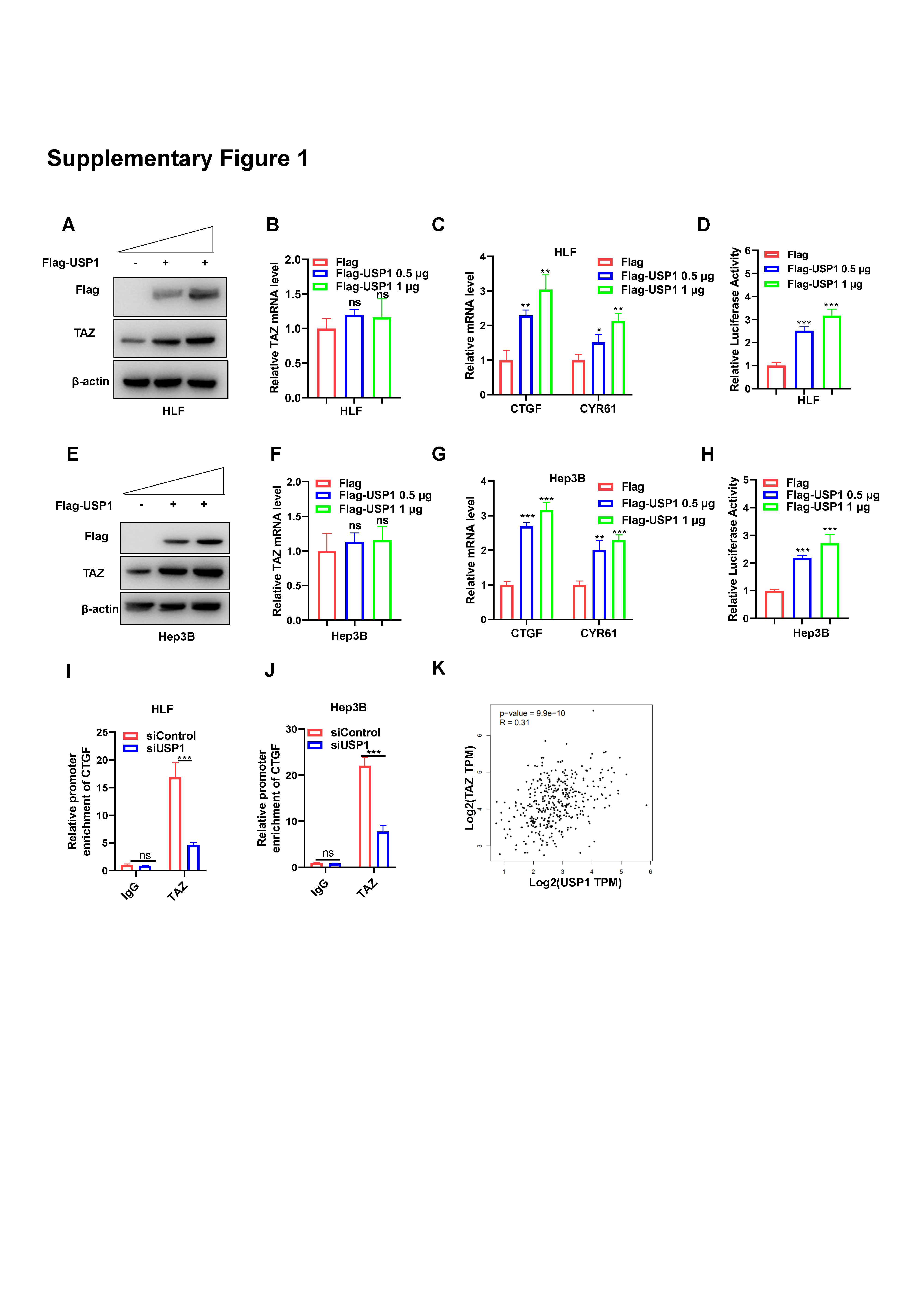

Supplement: Supplementary file 4 — supplementary figure 1 [file 41419_2023_5777_MOESM4_ESM.tif]

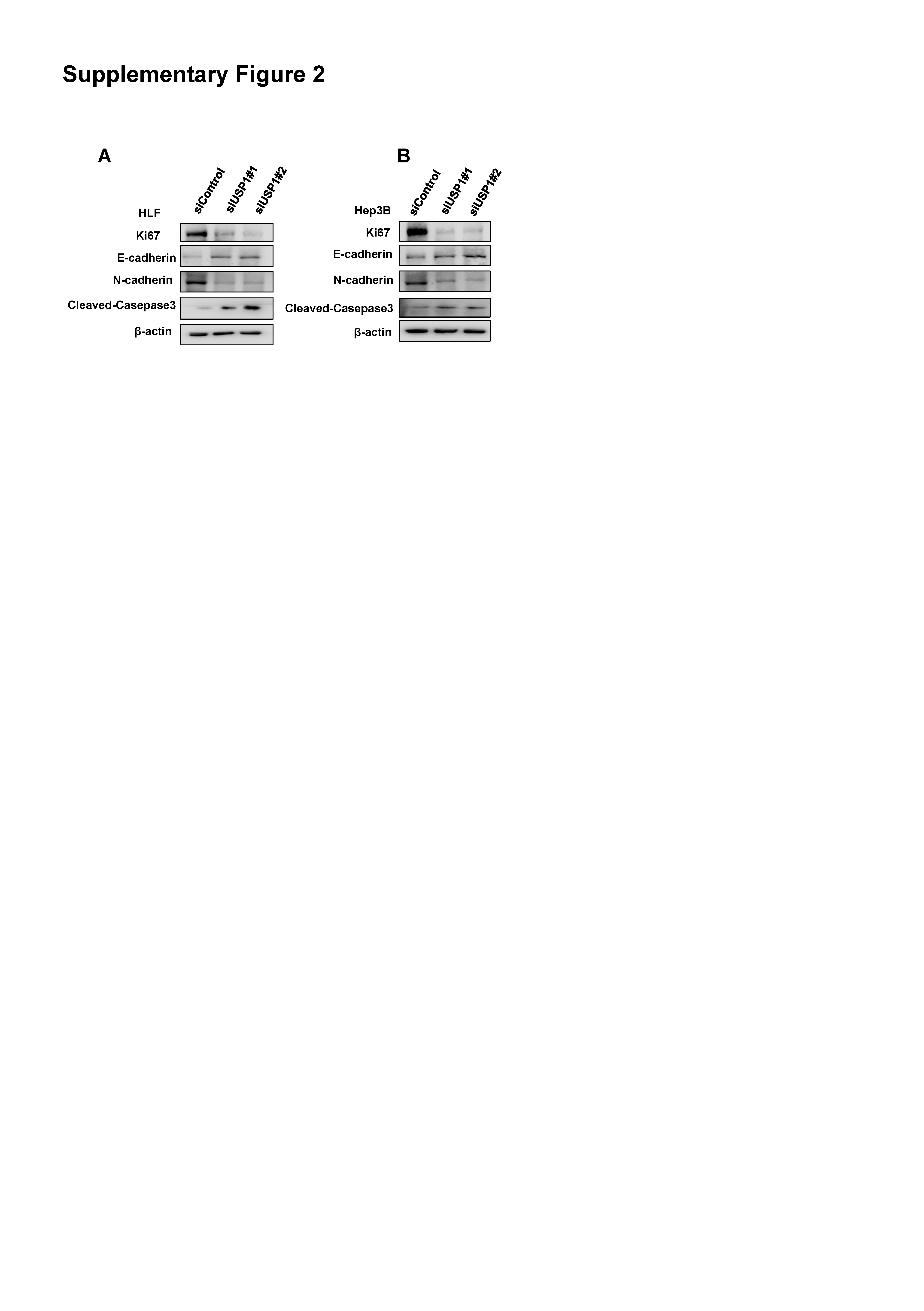

Supplement: Supplementary file 5 — supplementary figure 2 [file 41419_2023_5777_MOESM5_ESM.tif]

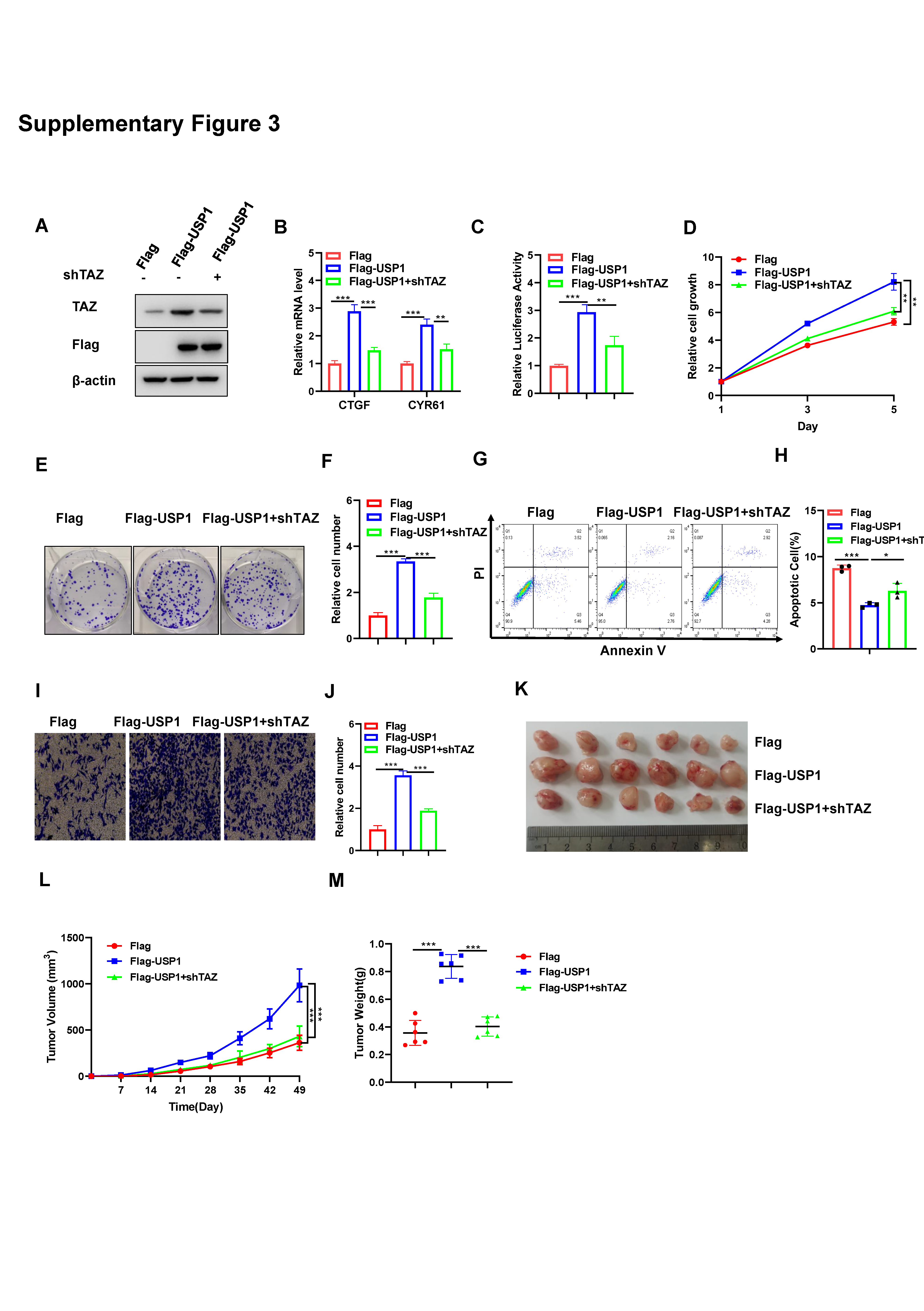

Supplement: Supplementary file 6 — supplementary figure 3 [file 41419_2023_5777_MOESM6_ESM.tif]
